# Morphology and properties of pyrite nanoparticles obtained by pulsed laser ablation in liquid and thin films for photodetection

**DOI:** 10.3762/bjnano.16.60

**Published:** 2025-06-03

**Authors:** Akshana Parameswaran Sreekala, Bindu Krishnan, Rene Fabian Cienfuegos Pelaes, David Avellaneda Avellaneda, Josué Amílcar Aguilar-Martínez, Sadasivan Shaji

**Affiliations:** 1 Facultad de Ingeniería Mecánica y Eléctrica, Universidad Autónoma de Nuevo León, San Nicolás de los Garza, Nuevo León, 66455, México; 2 Current affiliation: Instituto de Ciencias Aplicadas y Tecnología, Universidad Nacional Autonoma de Mexico, Circuito Exterior S/n, Ciudad Universitaria, Ciudad de Mexico, C.P. 04510, Mexico; 3 Centro de Innovación, Investigación y desarrollo en Ingeniería y Tecnología (CIIDIT), Universidad Autónoma de Nuevo León, PIIT Monterrey, Apodaca, Nuevo León, 66629, México; 4 Centro de Investigación en Innovación en Ingeniería Aeronáutica, Universidad Autónoma de Nuevo León, Carretera a Salinas Victoria K.M. 24.5, Apodaca, Nuevo León 66600, México

**Keywords:** electrophoretic deposition, pulsed laser ablation in liquid, pyrite nanoparticles, self-powered photodetector, spin coating

## Abstract

This work reports the synthesis of pyrite (iron disulfide (FeS_2_)) nanoparticles (NPs) of different morphologies using pulsed laser ablation in liquid (PLAL) in different organic solvents. The impact of the solvent on the morphological, compositional, and optical properties of the synthesized NPs is investigated by techniques such as transmission electron microscopy, scanning electron microscopy, X-ray photoelectron spectroscopy, and ultraviolet–visible spectroscopy. The morphology of the NPs in different solvents varied from spherical, rice-like to rod-like particles which demonstrates the effect of the solvent on the morphology/composition of NPs synthesized by PLAL. FeS_2_ NPs were successfully synthesized in five different solvents, along with a minor phase of iron sulfide (FeS). Additionally, by combining electrophoretic deposition and spin-coating techniques, thin film photodiodes of FeS_2_ were fabricated on an n-type Si substrate utilizing the nanocolloids. The structural, morphological and electrical characterizations of the films are also presented. By sulfurization of the films, phase-pure pyrite thin films are obtained. The photodetection range was up to 785 nm photocurrent in the order of 10^−6^ to 10^−4^ A for different annealing conditions and a detectivity in the order of 10^6^–10^8^ Jones is attained. The FeS_2_/n-Si photodetector works in self-powered mode also in addition to the photoconductive mode. The results show the effective fabrication of nanostructured ultraviolet–visible–near infrared thin film photodetectors using semiconductor nanocolloids prepared by PLAL.

## Introduction

Pyrite (FeS_2_) is one of the earth-abundant and nontoxic semiconductors possessing a promising role in optoelectronic applications. FeS_2_ has a narrow bandgap (0.95 eV), high light absorption coefficient (≈10^5^ cm^−1^), excellent properties in photoelectric conversion, and has enormous potential as an efficient photodetector system and in lithium batteries [1–2]. The prevalent forms of FeS_2_ are cubic-system pyrite and the orthorhombic-system marcasite crystal structure. Due to its low structural symmetry, marcasite FeS_2_ has a bandgap of only 0.34 eV and, as a result, it is not appropriate for use, particularly with solar energy absorption materials. Hence, the pyrite structure serves as a foundation for almost all studies of systems related to FeS_2_ [3]. Pyrite has shown outstanding performance and a long shelf life as a high-capacity cathode and has been utilized in batteries [4]. Pyrite has also been identified as a promising material for effectively removing environmental contaminants in the environment near the surface of the Earth, including toxic heavy metals and metalloids, radionuclides, and organic pollutants [5]. In addition to the aforementioned advantages as a semiconductor, it has the potential to be used in various applications through its nanostructures created via pulsed laser ablation in liquid (PLAL) and thin films.

The conventional methods reported for the synthesis of pyrite NPs include chemical methods using stabilizing agents [4], high-energy mechanical ball milling [6–7], colloidal pyrite by wet solution/phased chemical approaches [8–9], and hot injection [10–11]. In this study, PLAL was employed to synthesize pyrite nanoparticles in various solvents, including isopropyl alcohol (IPA), dimethyl formamide (DMF), ethanol, methanol, and acetone, using a 532 nm wavelength output from a Nd:YAG laser. Pulsed laser ablation in liquid has been demonstrated as a reliable alternative to conventional chemical reduction methods for the synthesis of NPs without the need for chemical reagents [12]. Laser ablation techniques are intrinsically efficient and require little manual labor and do not require extreme conditions, such as high vacuum, temperature, and pressure [13–15]. One of the many advantages of PLAL is that the productivity and morphology/size of the NPs generated can be regulated by carefully managing the input parameters [16]. Due to the challenges in obtaining phase-pure FeS_2_ by PLAL, this is a much less explored material despite its high potential. A strong reducible ferric ion and an oxidizable sulfide ion coexist in iron sulfides, making their synthesis more difficult than that of iron oxide [17]. Unlike FeS_2_, there are reports on the synthesis of other iron-based oxides via PLAL. These studies have demonstrated the successful production of Fe_3_O_4_ nanoparticles by ablating iron plates in liquid using different wavelengths and laser fluences [18]. During the synthesis of FeS_2_ NPs by PLAL in different solvents, Motohashi et al. has reported the formation of hematite (Fe_2_O_3_) as major product and Fe–S as minor product [19]. Sai et al. synthesized pyrite nanoparticles for photovoltaic performance by first fabricating amorphous iron oxide films on normal glass substrates by spray pyrolysis followed by heating in sulfur atmosphere at 350 and 400 °C [20].

For pyrite film fabrication, solvothermal or hydrothermal and chemical synthetic routes are generally adopted [21–23]. Henríquez et al. reported the synthesis of single-phase FeS_2_ thin films using a combination of electrochemical and hydrothermal techniques. The electrodeposition was performed in a nonaqueous electrolytic bath consisting of diethylene glycol [24]. Among the deposition techniques, electrophoretic deposition (EPD) is a cost-effective method in which charged particles, dispersed in an organic medium, migrate towards the countercharged electrode upon the application of voltage, resulting in the formation of a thin coating [25]. Due to the vast variety of dimensions in terms of thickness of the deposit that is created, EPD spans a large range of applications. This method does not require expensive equipment or expert labor, and the coatings produced are generally quite homogenous. Other benefits include quick deposition, no substrate shape constraint, application to any material that is available as a fine powder or charged colloidal suspension. The EPD process is far more adaptable than other cutting-edge shaping methods since it is simple to alter for a particular purpose. By straightforward adjustments of the deposition time and applied voltage, EPD allows simple control over the thickness and shape of deposited layers [26–28]. In previous reports on pyrite thin films produced by EPD, Duan et al. deposited pyrite powder created using the sol–gel hydrothermal technique onto ITO substrates [29]. There are no other reports on the deposition of pyrite films using electrophoretic deposition. Spin coating is another thin film deposition technique where a substrate surface can be uniformly coated by spreading an evenly distributed layer of a desired material ink (in this case, NPs in a solvent) across the surface of the rotating substrate [30]. With these benefits, EPD and spin coating can be described as straightforward, flexible, and affordable methods to obtain thin films using laser-processed nanocolloids. Spin coating is combined with EPD for film deposition with the intention of obtaining seeded film growth with improved thickness and for surface morphology modifications. The importance of using nanoparticles in photodetection is that the practical detection wavelength range for conventional photosensors may be efficiently tuned by the wavelength-shifting capabilities of nanoparticles, making them appealing candidates for photon detection. The dimensions of the NPs play a crucial role in the shift of such properties [31]. For instance, it has been demonstrated that the wavelength-shifting characteristics of Si nanoparticles were caused by the effects of quantum-size confinement. The bandgap of silicon increased from its typical 1.1 eV in elemental form to nearly 3 eV in nanoparticle form, enhancing its ability to absorb UV wavelengths [32]. This concept was used to fabricate photodetectors (PDs) using pyrite NPs on Si substrate as it inherently has the advantages of exceptional photo-absorption, high mobility, and high absorption coefficient as initially mentioned. Self-powering PDs have a number of benefits, including small size, light weight, affordable price, low power consumption, high photoresponsivity, quick response, and most importantly, it independently operates without an external power supply and relies on the built-in potential, which helps to save energy [33]. Moreover, self-powered PDs, as opposed to conventional PDs, are well suited to operate in challenging environments and their applications and benefits in fields such as wireless environmental sensing, chemical and biosensing, in situ medical-therapy monitoring are explained in detail by Xu et al. in their review [34].

In this work, surfactant-free pyrite nanoparticles are synthesized by employing laser ablation in liquid, with a minor phase of FeS, which originated from the ablation target. Upon film fabrication using laser-processed nanocolloids, pure FeS_2_ phase is obtained by a sulfurization process. Since PLAL results in obtainment of stable nanoparticles with highly active and pure surface in their colloidal form, electrophoretic deposition is an attractive technique for their thin film deposition. Electrophoretic deposition followed by spin-coating technique is applied for thin film fabrication using the nanocolloid in this work. The optical properties of nanocolloids and their thin films were evaluated using UV–visible (UV–vis) spectroscopy. The nanoparticle characterization and surface morphology were studied using transmission electron microscopy (TEM), scanning electron microscopy (SEM), and the crystalline structure of the films was characterized by X-ray diffraction (XRD). The UV–vis–NIR photodetection properties of p-n junction-based thin films composed of FeS_2_ nanoparticles are reported. Additionally, the results of various light-sensing parameters evaluated in these photodetectors are also presented. Photodiode configuration of n-Si/p-FeS_2_ is achieved using nanocolloids synthesized in IPA and DMF. Annealing of these structures is also done in vacuum at different temperatures to improve their film properties and device performance. The photodiodes prepared using thin films of these nanocolloids can be operated in self-powered mode.

## Synthesis and Characterization

### Synthesis of FeS_2_ nanoparticles

**Figure 1:**
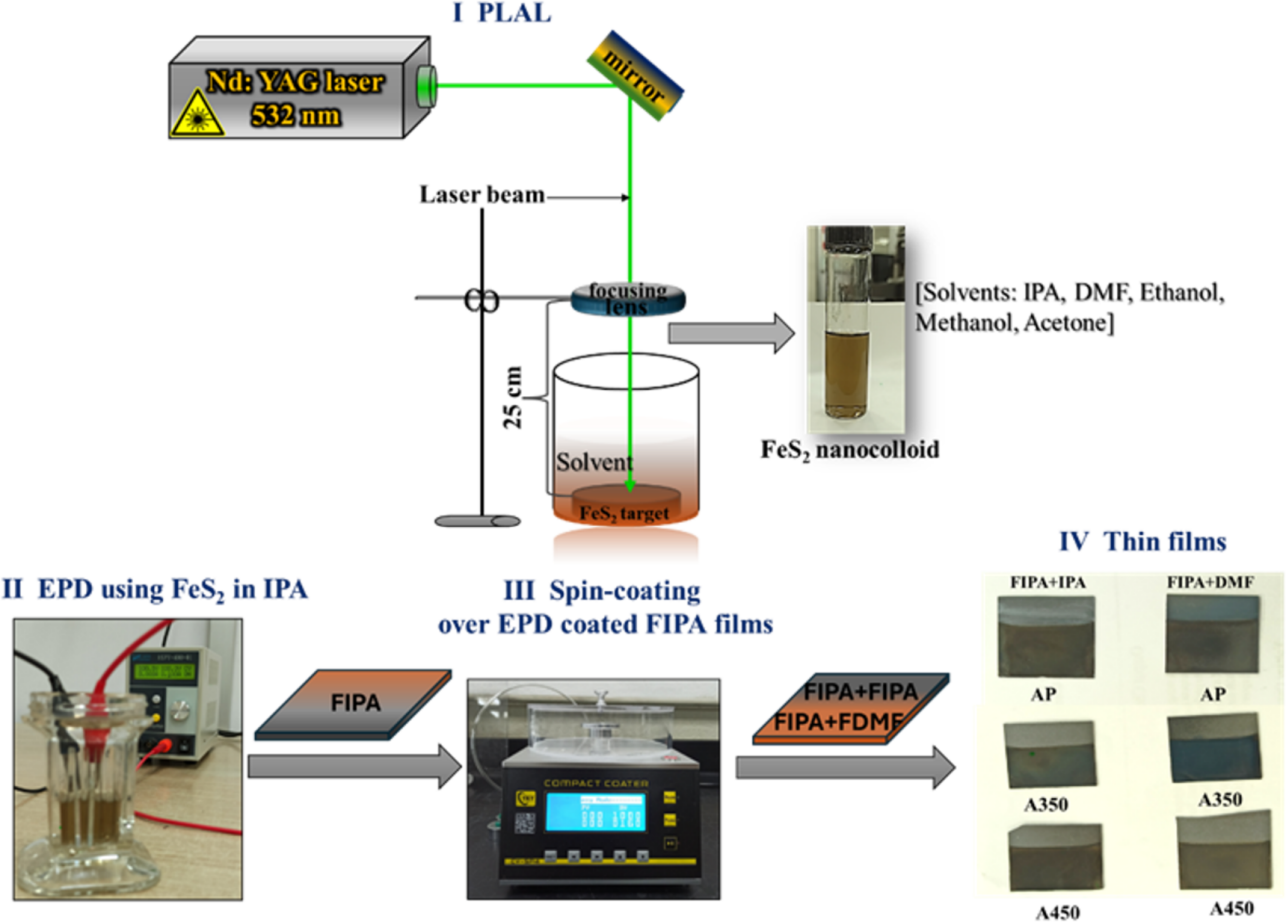
Schematic illustration of the methodology used for the synthesis of FeS_2_ nanoparticles by PLAL (I) followed by fabrication of their thin films by combining EPD (II) and spin coating (III). Images of as-prepared (AP) and annealed samples of FeS_2_ thin films (A350 and A450) are included (IV).

The schematic representation of the methodology utilized is given in Figure 1. FeS_2_ nanoparticles were synthesized using pulsed laser ablation in liquid. For laser ablation, the FeS_2_ target (99.9% pure, Beijing Goodwill Metals, China) was placed in a 250 mL beaker with 25 mL of solvent (IPA, DMF, ethanol, methanol, and acetone – all from Fermont, 99.9% pure, analytical grade). The FeS_2_ target surface was polished and washed each time using the respective solvent for ablation to get rid of any impurities on the target surface. The second harmonic output wavelength (532 nm) was employed to irradiate the FeS_2_ target positioned in the beaker in a vertical configuration of the experiment. For the ablation, a pulsed Nd:YAG laser (Model LQ 629, Solar Laser System) with pulse width of 10 ns, pulse repetition frequency of 100 Hz, and energy of 90 mJ/pulse was used. In each solvent, laser ablation was carried out for 10 minutes with the aid of a focusing lens (focal length of 50 cm) kept at a 25 cm distance from the target. The laser fluence calculated at this point was 0.23 J·cm^−2^. Images of the pyrite nanocolloids obtained in different solvents are shown in Supporting Information File 1, Figure S1. The NPs of FeS_2_ in IPA are referred to as FIPA, in DMF as FDMF, in ethanol as FET, in methanol as FMET, and in acetone as FAC. Morphology and composition of the nanoparticles obtained in all solvents are analyzed. Further, FeS_2_ nanocolloids in IPA and DMF (FIPA and FDMF) are used for thin films and photosensing device fabrication.

### Electrophoretic deposition of FeS_2_ nanoparticles

The electrophoretic deposition process was used for thin film fabrication using laser generated nanocolloids. Using the hot probe method [35], the p-type nature of FeS_2_ was identified. Based on this, an n-type Si (n-Si) substrate was chosen to achieve the photodiode structure. Well cleaned n-type silicon substrates were used as the anode and the cathode in the EPD setup. With a 4.5 mm gap between them, the electrodes were positioned parallel to one another and immersed in 12.5 mL FeS_2_ nanocolloid (FIPA) kept at room temperature during EPD. A potential difference of 400 V (constant voltage, with current of approximately 2 mA) was applied after optimization of EPD conditions. The deposition was carried out for 1 h. The positive electrode was coated with FeS_2_ nanoparticles.

### Spin coating of FeS_2_ nanoparticles

Following EPD, FeS_2_ nanocolloids in IPA and DMF were individually spin-coated onto thin films for seeded growth, improved thickness, and to avail the use of the morphological characteristics of FeS_2_ nanoparticles in both solvents. A spin rate of 400 rpm, an acceleration of 20 rpm/s, and a 20 s spin time were the conditions used for spin coating. The films formed by combining EPD and spin coating were annealed at 350 and 450 °C for 30 min in vacuum. The films under as-prepared and annealed conditions are given in Figure 1. The FIPA that was deposited by EPD and then spin coated with FeS_2_ NPs in DMF is indicated as "FIPA+DMF A350" in Figure 1. The as-prepared film is referred to as AP, and the annealing temperature is referred to as A350 and A450. A photodiode structure of p-FeS_2_/n-Si was successfully fabricated.

### Sufurization of films

For this experiment, FDMF was drop-casted onto FTO substrates (2.5 × 2 × 0.1 cm) and the film was annealed for 1 h at 300 °C in a sulfur environment. In a semi-cylindrical glass container (12 × 2.5 cm), 5 mg of sulfur powder was added, and samples were maintained closely to the powder. Aluminum foil was used to wrap the sample and the glass vessel before they were introduced into a quartz tubular furnace (Lindberg/Blue MTM Mini-Mite Tube Furnace), equipped with a temperature controller. At a very low-pressure, high-quality nitrogen gas was passed through the tubular furnace. The presence of nitrogen flow prevented the samples from oxidizing or being contaminated during sulfurization. The heated samples were gradually cooled down to room temperature. Throughout the experiment, the flow of nitrogen gas was kept steady. The optimal conditions were determined through trials using varying temperatures and amounts of sulfur.

### Characterization

The morphological analyses of pyrite NPs were recorded using the FEI Titan G2 80–300 for TEM, high-angle annular dark-field scanning transmission electron microscopy (HAADF-STEM), high-resolution TEM (HRTEM) and selected area electron diffraction (SAED). The SEM analysis of pyrite thin films was performed using a Hitachi Model SU 8020. The NPs were deposited on carbon-coated copper grids for TEM analysis and on silicon substrates for SEM analysis. Using monochromatic Al Kα radiation with an energy of 1486.68 eV, X-ray photoelectron spectroscopy (XPS, Thermo Scientific K-Alpha) was employed to characterize the elemental composition and chemical states of the elements in the NPs. Apart from using HRTEM and SAED for phase analysis, a Panalytical Empyrean Diffractometer (X-ray source of Cu Kα_1_ radiation = 1.54056 Å) was also used for X-ray diffraction analysis. A dual-beam UV–vis–NIR spectrophotometer was used to analyze the optical characteristics of the nanocolloids (Jasco V770). A Keithley 6487 picoammeter/voltage source was used to record the electrical properties of the films. The current–voltage (*I*–*V*) curves for the photodiodes were measured both in the dark and under illumination. Using silver paint (Flash-Dry silver colloidal suspension, SPI^®^ supplies), two contact electrodes with a 4 mm length and a 4 mm distance between them were prepared on the films for electrical measurements. The photoresponse curves were evaluated using light sources, including tungsten (W) lamps, LEDs (full spectrum, red, UV, and IR LEDs), and continuous-wave lasers of wavelengths 532 and 785 nm. The cyclic photoresponse of the samples to various light sources was also measured to determine their photostability.

## Results and Discussion

### Morphology of the pyrite NPs

**Figure 2:**
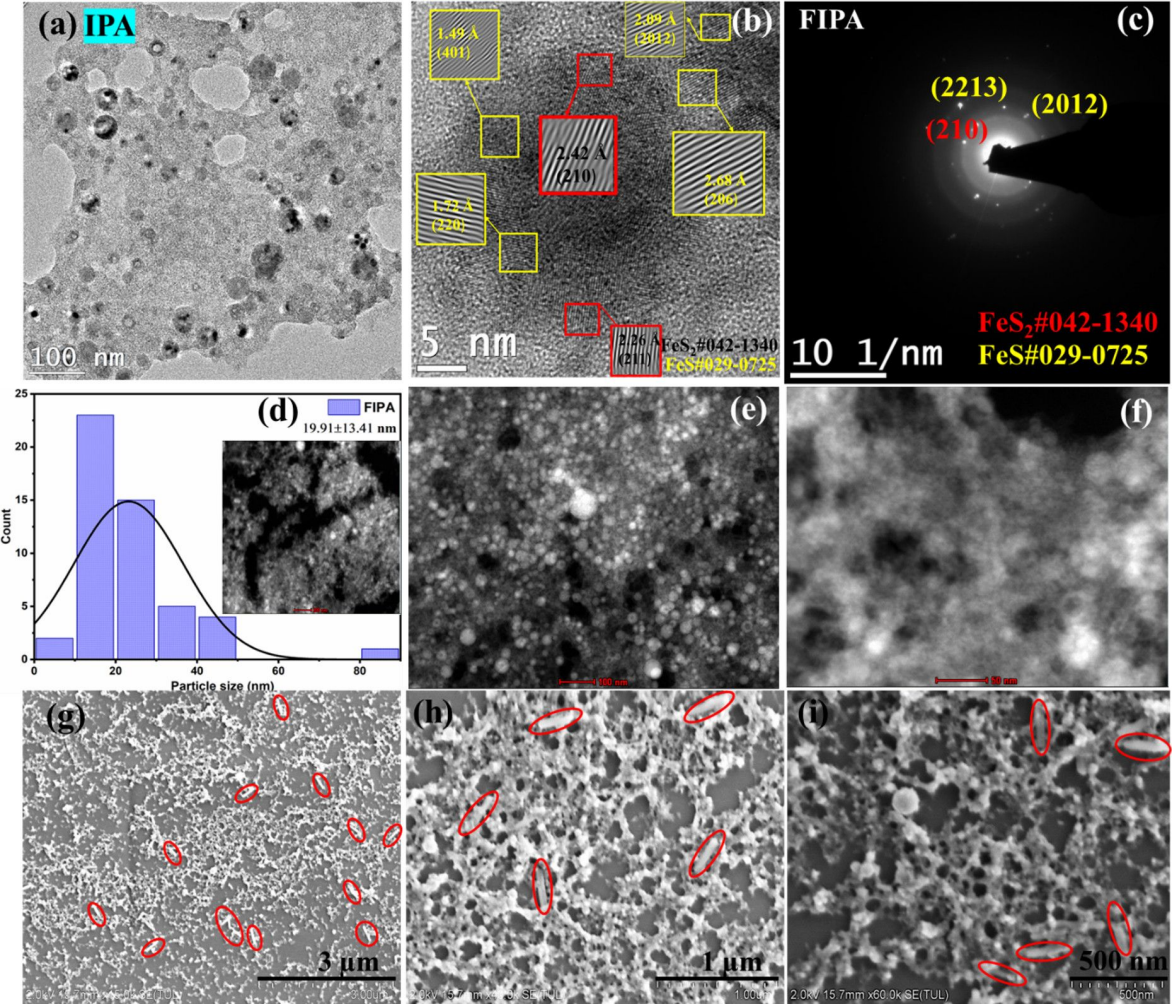
(a) TEM, (b) HRTEM, (c) SAED, (d) particle size distribution, (e, f) STEM (image scales 100 and 50 nm), and (g–i) SEM images (image scales are 3, 1 μm, and 500 nm) of FeS_2_ NPs in IPA.

**Figure 3:**
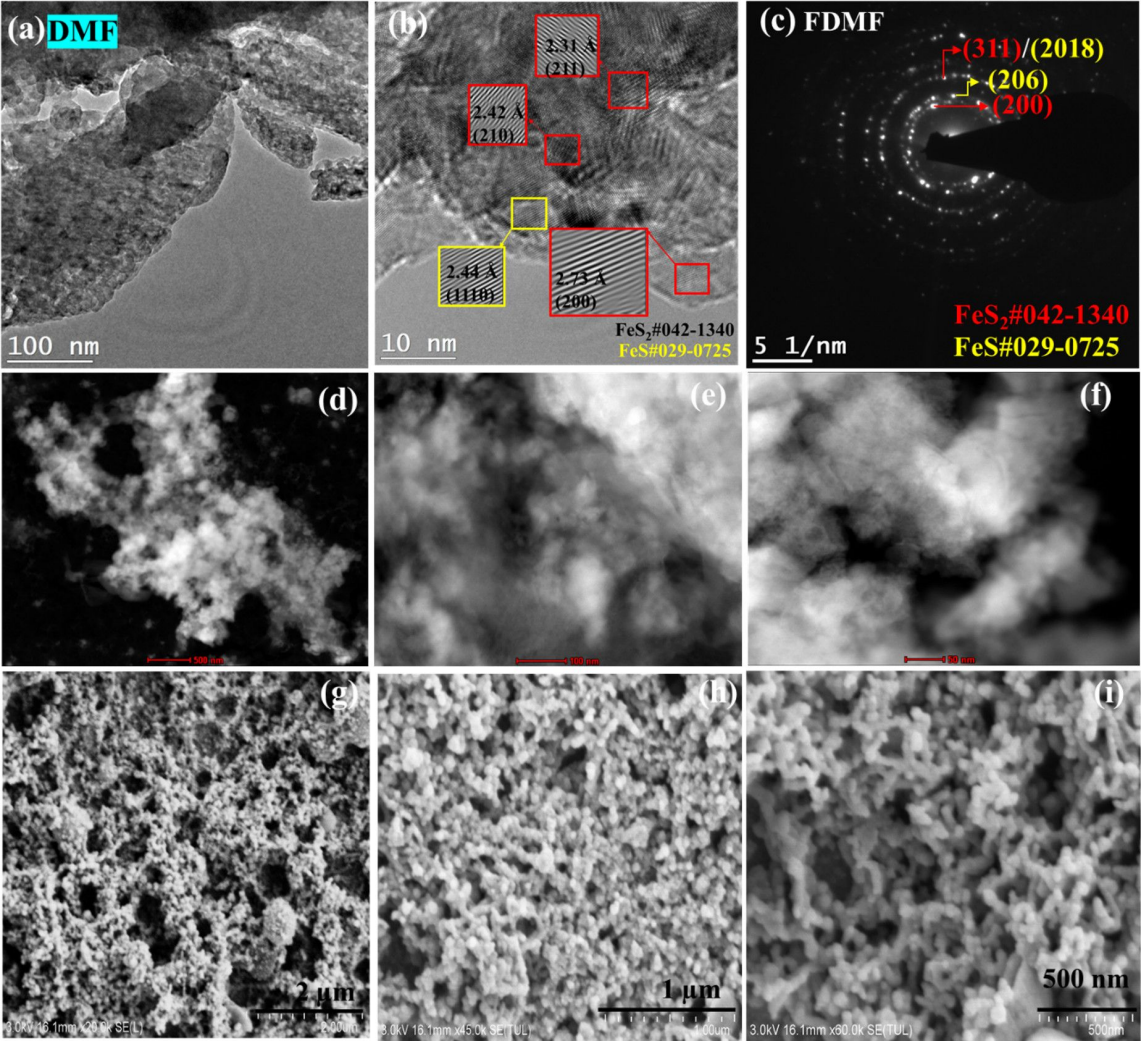
(a) TEM, (b) HRTEM, (c) SAED, (d–f) STEM (image scales are 500, 100, and 50 nm), and (g–i) SEM images (image scales are 2, 1 μm, and 500 nm) of FeS_2_ NPs in DMF.

**Figure 4:**
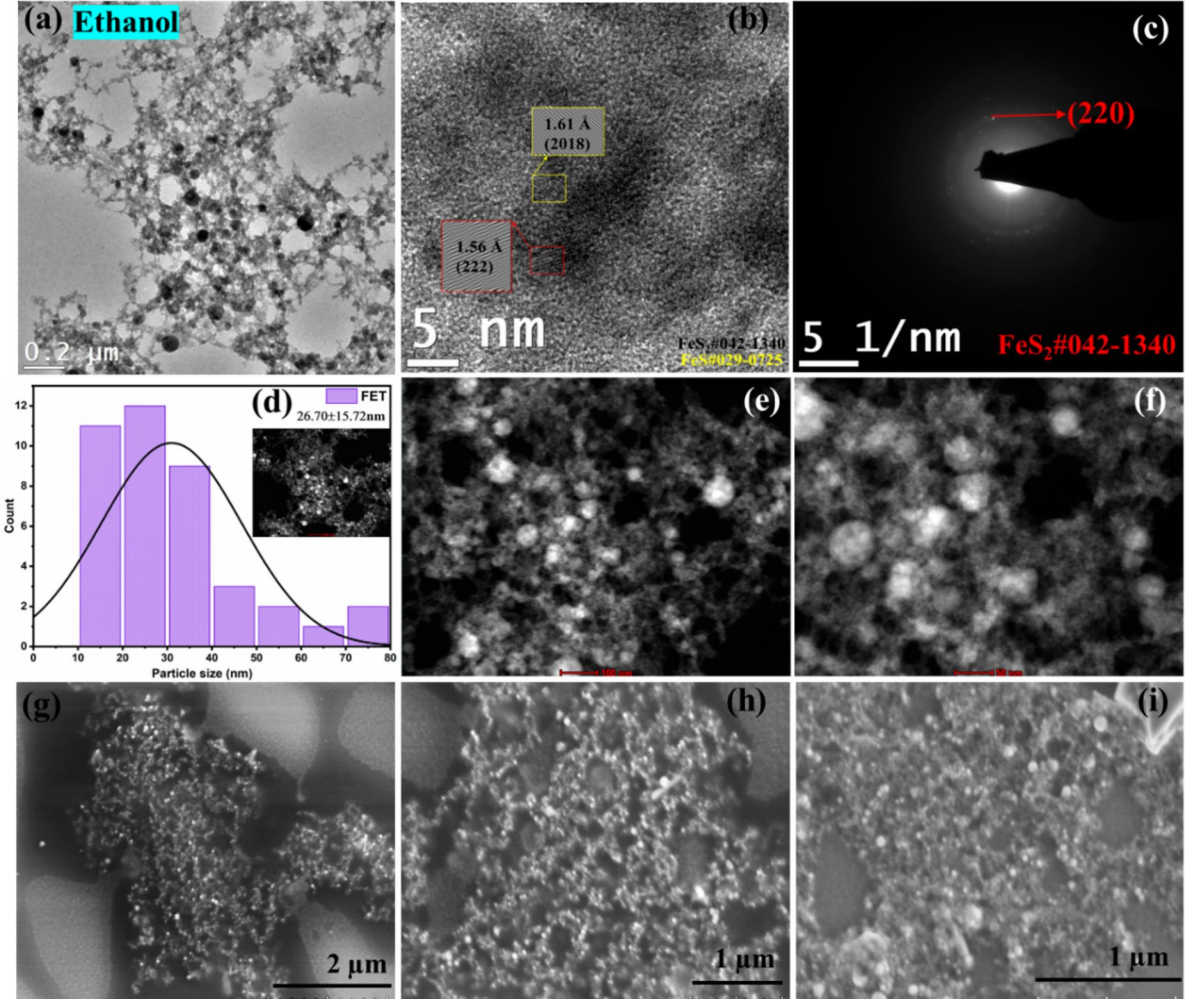
(a) TEM, (b) HRTEM, (c) SAED, (d) particle size distribution, (e, f) STEM (image scales 100 and 50 nm), and (g–i) SEM images (image scales are 2, 1, and 1 μm) of FeS_2_ NPs in ethanol.

**Figure 5:**
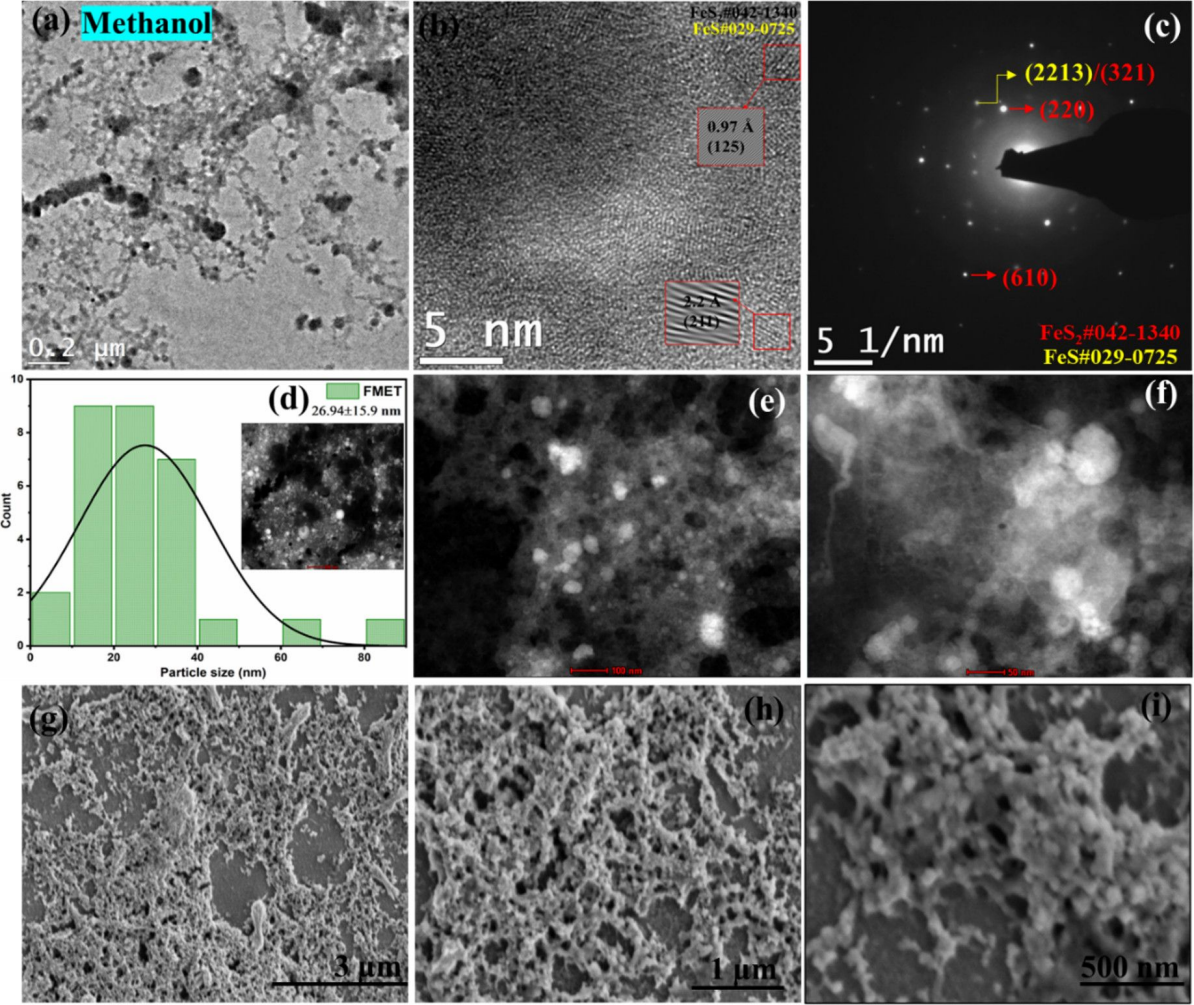
(a) TEM, (b) HRTEM, (c) SAED, (d) particle size distribution, (e, f) STEM (image scales 100 and 50 nm), and (g–i) SEM images (image scales are 3 μm, 1 μm, and 500 nm) of FeS_2_ NPs in methanol.

**Figure 6:**
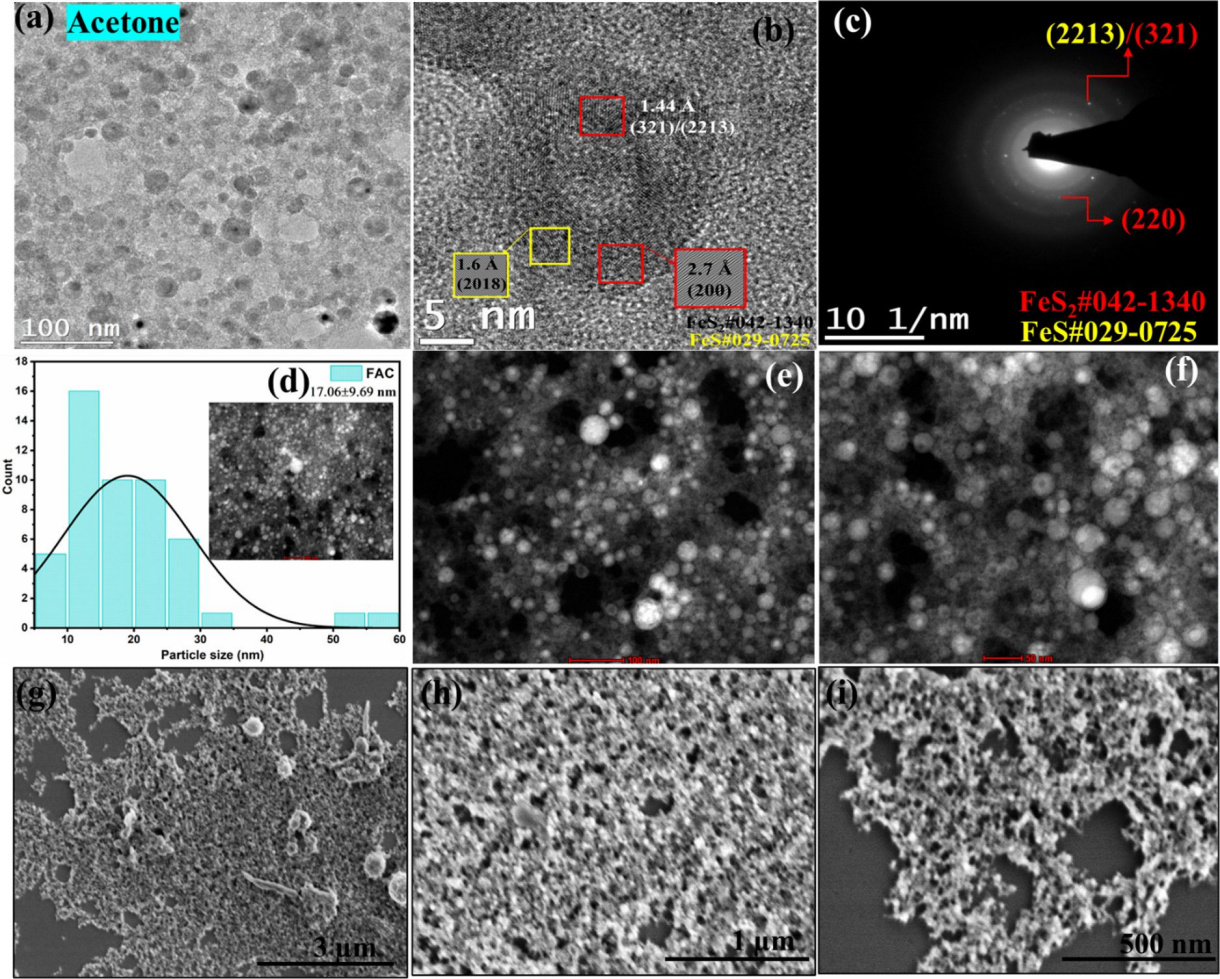
(a) TEM, (b) HRTEM, (c) SAED, (d) particle size distribution, (e, f) STEM (image scales 100 and 50 nm), and (g–i) SEM images (image scales are 3 μm, 1 μm, and 500 nm) of FeS_2_ NPs in acetone.

The morphological differences of the pyrite NPs generated in different solvents such as IPA, DMF, ethanol, methanol and acetone, and their respective TEM, HRTEM, and STEM images, particle size distribution, and SAED patterns are provided in Figures 2–6. The lattice fringes from HRTEM images and the diffraction spots from SAED patterns were indexed to that of the cubic phase of FeS_2_ (ICDD#042-1340) and hexagonal phase of FeS (ICDD#029-0725). The TEM, HRTEM, SAED, and STEM images of FIPA (FeS_2_ NPs in IPA) are shown in Figure 2a–f. FIPA exhibits spherical particles in TEM (Figure 2a) and STEM (Figure 2e,f) images but a combination of spherical and long rod-like particles are clearly seen in the SEM analysis. The rod-shaped particles highlighted in the SEM images are seen randomly distributed across some regions. On the other hand, most of the particles are spherical, which is consistent with what is observed in the TEM images. The rod-shaped particles, being fewer in number, are not captured in the selected TEM region. Also, in the TEM images of FIPA (Figure 2a), some hollow NPs are present. Hollow-structured NPs are typically formed through mechanisms such as the Kirkendall effect [36]. This effect arises when there is a difference in diffusion rates of the species in the core and shell, leading to the formation of voids within the particle. In our case, there is no presence of core–shell structure but the high viscosity of IPA likely plays a key role in the formation of these hollow structures. Specifically, larger-sized NPs tend to remain near the focused laser beam due to the high viscosity of the IPA during ablation. These larger particles interact with the prevailing laser pulses [13,37]. During this interaction, solid NPs break down into smaller-sized particles, which then contribute to the formation of hollow structures. This process is influenced by laser ablation conditions and physical properties of the surrounding liquid, similar to the mechanism described in the referenced article. Though the average NP size calculated from STEM images for FIPA NPs was 19.91 nm with a standard deviation of 13.41 nm, a few clusters were also observable and can be justified based on the bimodal size distribution of the particles (1 to 50 nm and from 80 to 85 nm) observed. The long rod-like particles in the SEM images (Figure 2g–i) are indicated by red oval shapes. The (210) and (211) planes that correlate to pyrite are identified in HRTEM with interplanar distances of 2.42 and 2.26 Å, respectively, as in Figure 2b. The planes corresponding to FeS are also found at interplanar distances of 1.49, 1.73, 2.09, and 2.68 Å, corresponding to the (401), (220), (2012), and (206) planes, respectively. From the diffraction spots of the SAED images (Figure 2c), both these phases are also identified. The (210) plane of FeS_2_ is detected at 2.42 Å, while the (2213) and (2012) planes of FeS are calculated at distances of 1.44 and 2.07 Å, respectively. Minor presence of FeS was identified in the target analyzed by XRD (Supporting Information File 1, Figure S3a).

Ultrafine spherical nanoparticles as condensed clusters are observed in TEM (Figure 3a) and STEM (Figure 3d–f) images for FeS_2_ NPs in DMF (FDMF). In comparison to IPA, a completely different type of morphology is observed for NPs in DMF from SEM, as images shown in Figure 3g–i. Only small spherical particles combined together to form long chains are seen. There was no presence of rod-shaped or extended particles at all. Also in the case of FDMF NPs, the HRTEM and SAED patterns (Figure 3b,c) show mixed phases of FeS_2_ and FeS. From HRTEM, (211), (210), (200) planes with d-spacing values of 2.31, 2.42, and 2.73 Å for pyrite; and (1110) plane at 2.44 Å for FeS are identified and marked in the images. From SAED, a calculated interplanar distance of 1.62 Å can be attributed to (311) or (2018) planes of pyrite or FeS respectively, and 2.7 Å corresponds to (200) plane of FeS_2_. The (206) plane of FeS at 2.65 Å is also identified. In the case of TEM and STEM images of NPs in DMF (Figure 3a,d–f), the majority of the analyzed regions resemble agglomerated ultrafine powders. This agglomeration may be attributed to the higher boiling point of DMF (153 °C), which causes it to evaporate more slowly, allowing more time for the particles to aggregate. In contrast, alcohols (boiling point range: 64–78 °C) and acetone (56 °C) evaporate much more quickly, significantly reducing the influence of the solvent and leading to less agglomeration in the samples prepared with these solvents for TEM analysis (Figure 4–6). As a result, although nanopowder-like particles are identified, the morphology of NPs in DMF from TEM and STEM images is not easily discernable.

For NPs in ethanol and methanol, interconnected spherical particles are observed, whereas for NPs in acetone, well-defined and much bigger spherical particles are formed. The TEM and SEM images are given in Figures 4, 5, and 6 respectively. In all these cases, the HRTEM, SAED, and STEM images are also analyzed. The (220) plane of FeS_2_ at a d-spacing value of 1.9 Å is identified from SAED patterns in all three cases. In addition, for NPs in methanol, (610) plane at a distance of 0.89 Å also corresponds to FeS_2_. For both methanol and acetone, (321)/(2213) planes of FeS_2_ and FeS at a distance of 1.44 Å are identified from SAED. From the HRTEM images of ethanol, (222) plane of pyrite (d-spacing: 1.56 Å), and (2018) plane of FeS (d-spacing: 1.61 Å) are identified. For NPs in methanol, (125) plane at 0.97 Å and (211) at 2.2 Å are found. In addition to the planes identified from SAED, (200) plane of pyrite at 2.7 Å is also seen from HRTEM of NPs in acetone. Interestingly, while core–shell NPs with hematite as the major phase were reported for laser ablation of pyrite in ethanol and acetone [19], our PLAL synthesis resulted in a completely different morphology, without the presence of any oxide phases. Yuan et al. reported morphologies of pyrite crystals such as rod-like, cubic, and flake-like structures synthesized in aqueous solutions by chemical methods. The diverse range of observed pyrite morphologies highlights the influence of synthesis conditions on the final crystal shape [2]. In another study, pyrite nanoparticles (FeS_2_) were synthesized in deionized water with and without the assistance of a magnetic field by laser ablation in liquids. SEM and TEM images of FeS_2_ nanoparticles prepared without a magnetic field revealed spherical particles with varying sizes, aggregation and agglomeration with an average size of approximately 40 nm [38]. In contrast to this**,** the average particle sizes calculated in this work were 26.7, 26.9, and 17.1 nm for FeS_2_ NPs in ethanol, methanol, and acetone, respectively. A clear effect of different solvents on the morphology of the generated NPs by PLAL is visible from the TEM and SEM analyses in the current work. The histogram for size distribution and the standard deviation values in each case are given in the respective figures (Figures 4–6d).

From the above analysis, it is evident that different morphologies of FeS_2_ nanoparticles are generated by PLAL as the liquid medium changes. The ablation and nanoparticle formation mechanism begins with the FeS_2_ target absorbing the laser pulse energy and forming a plasma plume that is confined by the liquid. This is followed by the expansion of the plasma plume carrying the ablated material into the surrounding liquid and the production of a shockwave. The breakdown-related expansion of plasma pushing the surrounding liquid results in a cavitation bubble which is a thin layer of vapor of the surrounding liquid, as well as some material evaporating from the target. Some of the crucial elements influencing the formation of the cavitation bubble are the liquid environment, a stronger confinement of the plasma, and rapid energy transfer from the plasma to the surrounding liquid. The target and liquid are heated during the shockwave propagation, which may aid in the separation of material from the crater. The plasma plume cools down and releases energy to the liquid solution as it expands. This event causes the release of a second shockwave, which causes the cavitation bubble to expand in the liquid before collapsing on a time period of hundreds of microseconds releasing NPs in the liquid resulting in stable colloidal solution [15,39].

Despite the proposed laser ablation mechanism, there are experimental variables that cause the kinetics of the nanoparticles formation to change, resulting in nanoparticles with different sizes and morphologies [40]. Thermodynamic properties such as density, dielectric constant, viscosity, vapor pressure, and optical properties of the solvents are some of the factors that influence the mechanism of nanoparticle formation. The physical properties of all solvents are tabulated in Supporting Information File 1 (Table S1). Modifying the liquid medium is one of the most effective and adaptable techniques to control particle size distribution and morphology during nanoparticle synthesis by PLAL. For instance, it has been reported that the density and viscosity of the surrounding liquid influence the expansion of the plasma plume. The expansion dynamics of the plume differ due to the larger opposing force induced by the increased viscosity of the solvent, which in turn has an impact on the homogeneity of the plume [41]. Similarly, the average size of the nanoparticles shrinks as the dipole moment of the liquid medium rises. An electrical double layer is strongly formed on the surface of the nanoparticle due to a higher electrostatic interaction caused by the increased dipole moment. The interaction between nanoparticles and the plume depends on all attractive and repulsive forces, including the attractive van der Waals forces which cause growth and aggregation, and the electrostatic repulsive forces that are generated as a result of the overlap of electrical double layers [42]. Since acetone (2.88 *D*) and DMF (3.86 *D*) have greater dipole moments than alcohols (1.69 D), the size of FeS_2_ NPs produced by ablation in these solvents are expected to be smaller [43]. Results obtained in this study corroborate this finding. The diameters of NPs in IPA, ethanol, and methanol are 8–85 nm whereas those in acetone range between 6–55 nm.

### Elemental composition analysis of the NPs

**Figure 7:**
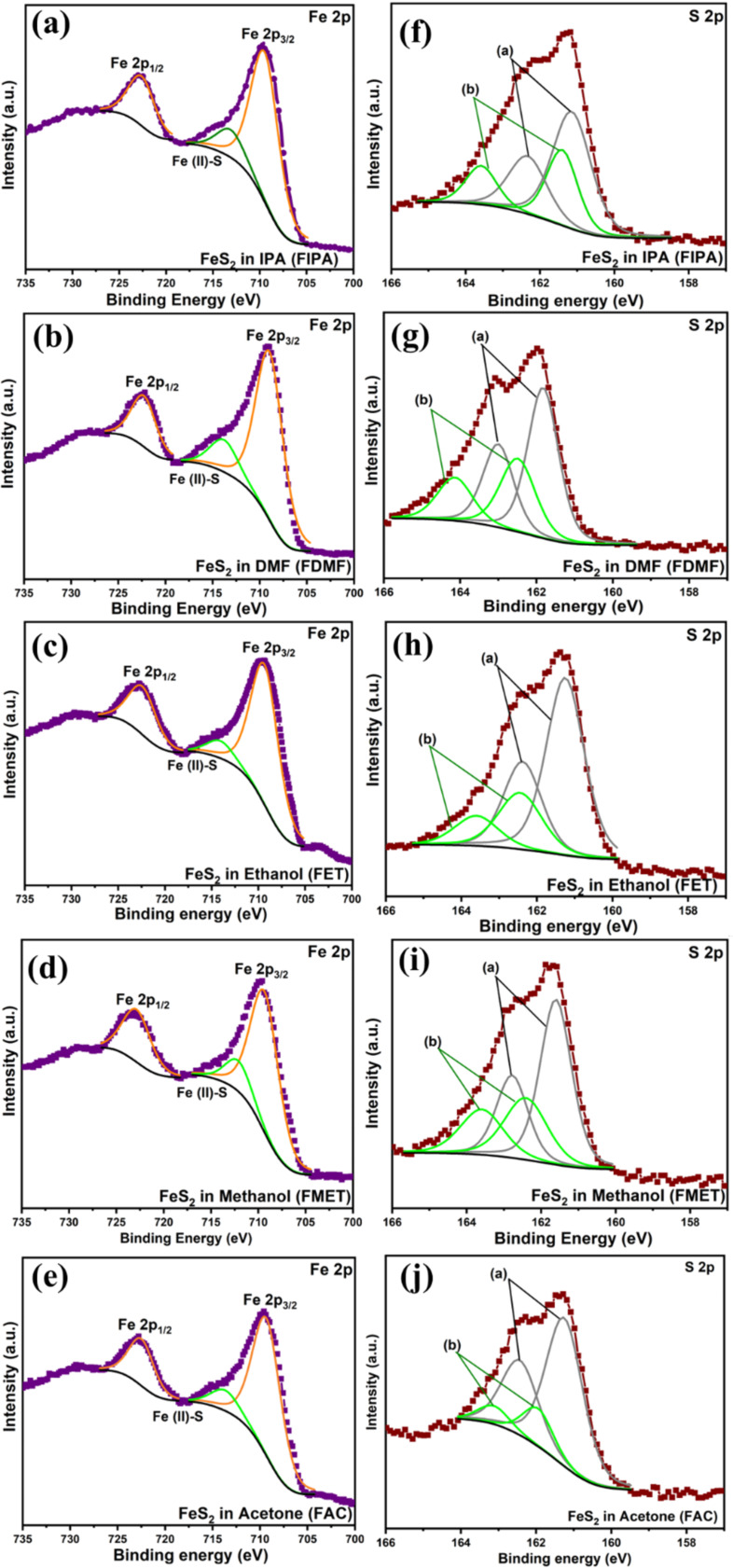
High-resolution XPS spectra of (a–e) Fe 2p, and (f–j) S 2p of FeS_2_ NPs in different solvents (FIPA, FDMF, FET, FMET, and FAC).

By drop-casting these pyrite NPs onto FTO substrates and analyzing them using XPS, the elemental composition of the particles in all the solvents was identified. For binding energy correction of all the samples, the peak position corresponding to the adventitious carbon value was fixed at 284.6 eV. A Shirley-type background baseline was used, and the Gaussian–Lorentzian sum function was applied for peak fitting. The high-resolution spectra of Fe 2p and S 2p after soft surface etching using argon ions is provided in Figure 7. The Fe 2p spectra of pyrite NPs in all solvents (Figure 7a–e) show two major peaks of Fe 2p_3/2_ and Fe 2p_1/2_ at 709 and 722 eV, respectively, which denotes the Fe^2+^ chemical state [44] with a separation Δ*E* of 13.1 eV. The peak fitting of the Fe 2p_3/2_ peak reveals a small peak around 713–714 eV associated to the Fe(II)–S bond of FeS [45], which is consistent with the small amount of FeS present in the film. No significant satellite peaks were observed near Fe 2p_1/2_, implying only a small quantity of the FeS phase or the dominance of Fe^2+^ in FeS_2_ in the films, and the peak fitting is in accordance to the hybrids reported [46–47]. The Fe 2p_1/2_ peak does not require deconvolution due to its relatively lower intensity and minimal contribution to the overall spectral profile. Specifically, in studies of iron, as proposed by Grosvenor et al., the Fe 2p_3/2_ peak needs fitting as it exhibits broadening in high-spin Fe^3+^ and Fe^2+^ compounds compared to Fe(0) metal or low-spin Fe^2+^ [48]. In our analysis, where pyrite (FeS_2_) is a high-spin Fe^2+^ complex due to the weak field ligand (sulfur) around the Fe^2+^ ion, a fitting approach similar to the one outlined in their work is followed. The S 2p peaks in all the samples were deconvoluted to S 2p_3/2_ and S 2p_1/2_ whose intensity was maintained at 2:1 with a binding energy difference of 1.18 eV between the peaks (Figure 7f–j). The peaks at 161 eV (S 2p_3/2_a) and 162 eV (S 2p_1/2_a) are the contributions of the S 2p spin orbital of S^2^**^−^** in FeS [49]. The bulk core level of S bound as a disulfide group (S_2_^2^**^−^**) in iron pyrite, S 2p_3/2_b and S 2p_1/2_b, respectively, was centered around 162 and 164 eV. To form S–S dimers, each S atom forms a tetrahedrally coordinated bond with three Fe^2+^ and one other S atom [50–52]. The exact binding energy values detected for Fe and S identified from high resolution spectra of Fe 2p and S 2p are given in Supporting Information File 1, Table S2.

### Optical properties of NPs and thin films

Supporting Information File 1, Figure S2a shows the UV–vis spectra of FeS_2_ nanoparticles in different solvents. The absorption onset of the NPs was around 400 nm except for NPs in ethanol and acetone. The slight variations in the optical absorption edges are results of differences in the shapes and sizes of the NPs generated in different liquid media [43]. The bandgap of the NPs is calculated using a Tauc plot and is provided in Supporting Information File 1, Figure S2b. For NPs in IPA, DMF, methanol, and ethanol, the values of bandgap were 3.01, 3.12, 3.06, and 3.6 eV, respectively. A bandgap of 4.36 eV has been reported for FeS_2_ NPs prepared by chemical methods [17]. Depending on the shape and size of the pyrite nanoparticles, bandgap values ranging from 0.90–3.1 eV are reported [53]. The variations in optical absorption edges and bandgaps may have been caused by different morphologies, particle sizes, and a minor presence of other phases. As the films were fabricated on an n-Si substrate which has a very reflective surface, the percentage reflectance was measured and is given in Supporting Information File 1, Figure S2c. Estimating the bandgap values of the films was challenging due to light scattering [8]. For homogenous and smooth deposition by EPD, it is crucial that the particles in the colloid are stable for a long time and thoroughly dispersed. Therefore, the NPs of FeS_2_ in ethanol and acetone were not attempted for EPD. Only NPs on IPA were employed for the initial research on film fabrication via EPD, followed by spin coating of NPs of DMF.

### X-ray diffraction studies of the films

**Figure 8:**
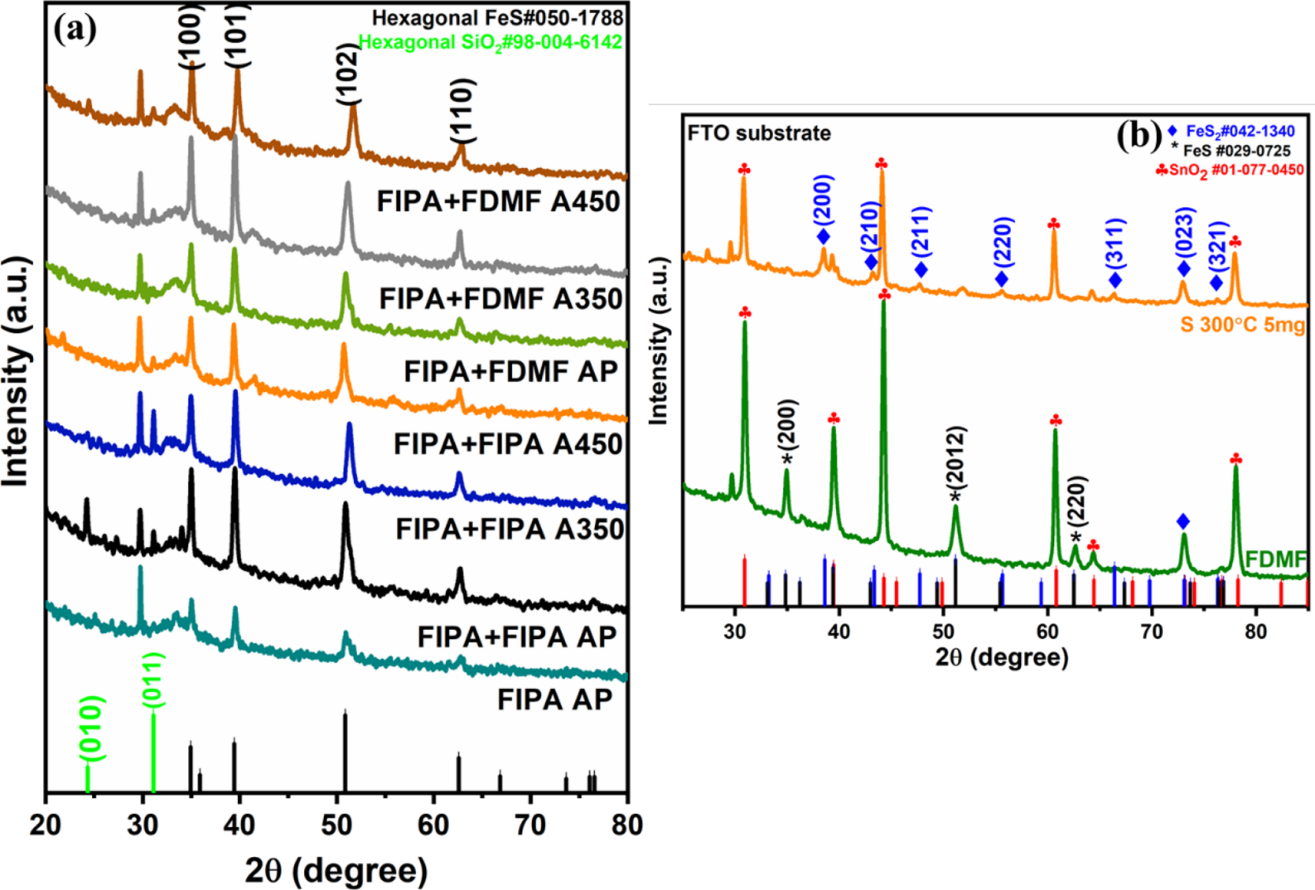
XRD patterns of (a) thin films of FeS_2_ fabricated on n-Si by EPD followed by spin coating (b) FDMF films on FTO after sulfurization.

XRD was utilized to identify the phases present in the FeS_2_ target that was used for laser ablation to produce nanocolloids, as well as to analyze the films deposited from these nanocolloids onto n-Si by EPD and spin coating. The cubic phase of pyrite (FeS_2_) and the hexagonal phase of pyrrhotite (FeS) are used to index all the diffraction peaks of the target (Supporting Information File 1, Figure S3a), which is consistent with the value provided in the standard cards, ICDD# 042-1340 and 029-0725 respectively. The refined scale factors are used to directly determine the phase abundances in the target and were calculated as 84.3% and 14.5% for pyrite and FeS, respectively. In the case of thin films, most of the peaks identified are those of FeS. The XRD patterns of the films on n-Si are given in Figure 8a. To confirm whether FeS is formed during the laser ablation process, the nanocolloids obtained after laser ablation in IPA and DMF (FIPA and FDMF) were drop casted onto FTO substrates and their XRD were analyzed, as shown in Supporting Information File 1, Figure S3b. FeS_2_ and FeS phases were also identified in the cases of drop-casted FIPA and FDMF films. Other peaks observed match those of the FTO substrate that was utilized for drop casting. Thus, it is presumed that the conversion of pyrite to FeS happens during the process of laser ablation itself. Although FeS was detected alongside FeS_2_, the XPS results indicate the FeS_2_ dominance, and the XRD analysis also validates it. A plausible mechanism is suggested for the formation of FeS. First, the FeS_2_ target may effectively absorb the laser light upon ablation, reach a high temperature, and split into Fe^2+^ and S_2_^2−^ ions. The presence of FeS in the target is also another reason for favoring this phase change. In the meantime, the surrounding solution (IPA or DMF, the solvent used) is rapidly heated and causes the partial reduction of S^1−^ (in S_2_^2−^) to S^2−^. The ions in the high-temperature bubble might have reacted quickly to form FeS nanoparticles. Zhou et al. explains a similar mechanism while describing the formation of Cu_1.4_S from Cu(acac)_2_ by laser ablation in liquid. In that case, the S^2−^ ions formed from the decomposition of an organic solvent (DMSO), Cu^2+^ formed by breaking down of Cu(acac)_2_, and Cu^+^ (reduction of Cu^2+^ in ethanol) reacted to form Cu_1.4_S NPs [54].

For obtaining phase-pure FeS_2_ films, a sulfurization method was applied. By experimenting with different sulfurization temperatures (from 200 to 300 °C) and amounts of sulfur powder (5 to 20 mg), the optimum condition for the formation of pure FeS_2_ phase was obtained. Using 5 mg of sulfur powder at 300 °C for 1 h, all the phases were converted to FeS_2_. Upon sulfurization, the color of the films turned grey. Figure 8b shows the XRD patterns of the films after sulfurization at 300 °C using 5 mg of S powder. For the pyrite FeS_2_ to be produced with precise stoichiometry, post sulfurization is necessary. The post-sulfurization process suppressed the secondary phases [55].

### SEM of the pyrite films

**Figure 9:**
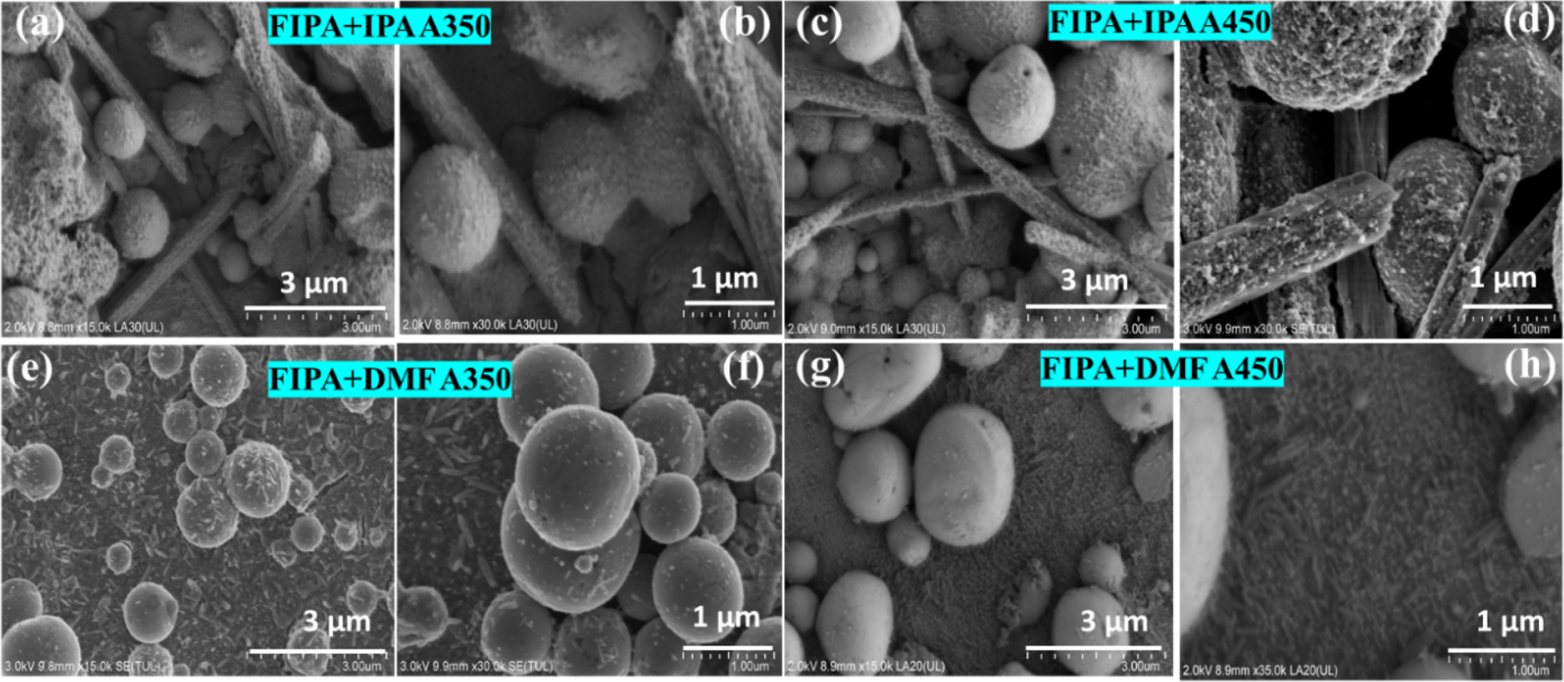
SEM images of annealed thin films fabricated on n-Si by EPD above which (a–d) FeS_2_ NPs in IPA (FIPA) and (e–h) DMF (FDMF) are spin coating.

Electrophoretic deposition with FeS_2_ nanoparticles in IPA (FIPA) on the surface of the Si substrate resulted in a relatively dense and compact formation, with particles closely packed together, forming a uniform and continuous film. The morphology of FeS_2_ NPs in EPD films retained their original morphology as observed in the colloidal state, with aggregates of spherical particles. Further control over film uniformity, packing density, or morphology can be achieved by modifying the deposition parameters in EPD, including voltage and deposition time [56]. The surface morphology analysis of the films fabricated by EPD and spin coating revealed that the morphology is influenced by the solvent used. Specifically, films spin coated with FeS_2_ NPs in IPA (FIPA) exhibited both spherical and elongated rod-shaped particles, with relatively uniform surface coverage. In contrast, films spin coated with FeS_2_ NPs in DMF (FDMF) exhibited only spherical particles with rice-shaped morphologies and less uniform coverage. Figure 9a–d shows the micrographs of FIPA spin-coated films, and Figure 9e–h shows the FDMF-coated samples under different annealed conditions. The SEM micrographs of the as-prepared films is given in Supporting Information File 1, Figure S4. These differences are likely attributed to variations in solvent properties and particle interactions during deposition. The morphologies of the FeS_2_ thin films vary depending on the different synthesis and fabrication techniques; hence, completely different morphologies are reported. Zebarjad et al. reported cauliflower-like blotches developed on the rough surface of nanostructured FeS_2_ films made via electrodeposition [57]. Nanowires, nanorods, and nanoribbons of 1D single crystalline FeS_2_ nanostructures with pyrite phase has been produced by experimenting on the concentration of iron source, temperature, and molar concentrations of the precursors in the solvent while synthesising using the solvothermal method [58]. As seen in our study, the surface of the films was covered by the morphology dominant from the NPs used for spin coating. For instance, when FIPA was used, spherical and rod-shaped particles were present, similar to the morphology observed in the nanocolloid. Conversely, spin coating with FDMF resulted in spherical particles with rice-shaped morphology. In a study on the synthesis of nanostructured powders and thin films of iron sulfide from molecular precursors by spin coating technique [59], clusters of densely packed crystallites and spherical crystallites were observed. They concluded that morphology is influenced by temperature and precursor type. Furthermore, different morphologies such as cubic, tetrakaidecahedron, hollow sphere, and rod-like have been reported for different phases of FeS_2_ samples prepared in water by single-step hydrothermal process [2]. The transition from spherical to hollow spheres composed of rod-like structures was associated with the conversion of the marcasite phase into the pyrite phase.

The SEM images of as-prepared FDMF films on FTO after sulfurization at 300 °C using 5 mg S powder is given in Supporting Information File 1, Figure S5c,d. The presence of distinct and uniformly distributed spherical particles can be observed. For direct comparison, the images of the film (FDMF) before sulfurization is also presented (Supporting Information File 1, Figure S5a,b). Umehara et al. has reported the effect of sulfurization temperatures and observed that temperatures above 500 °C are not preferable for device fabrication processes due to roughnened surfaces and marcasite phases fomed at higher temperatures [60].

### Photodetection studies of films

**Figure 10:**
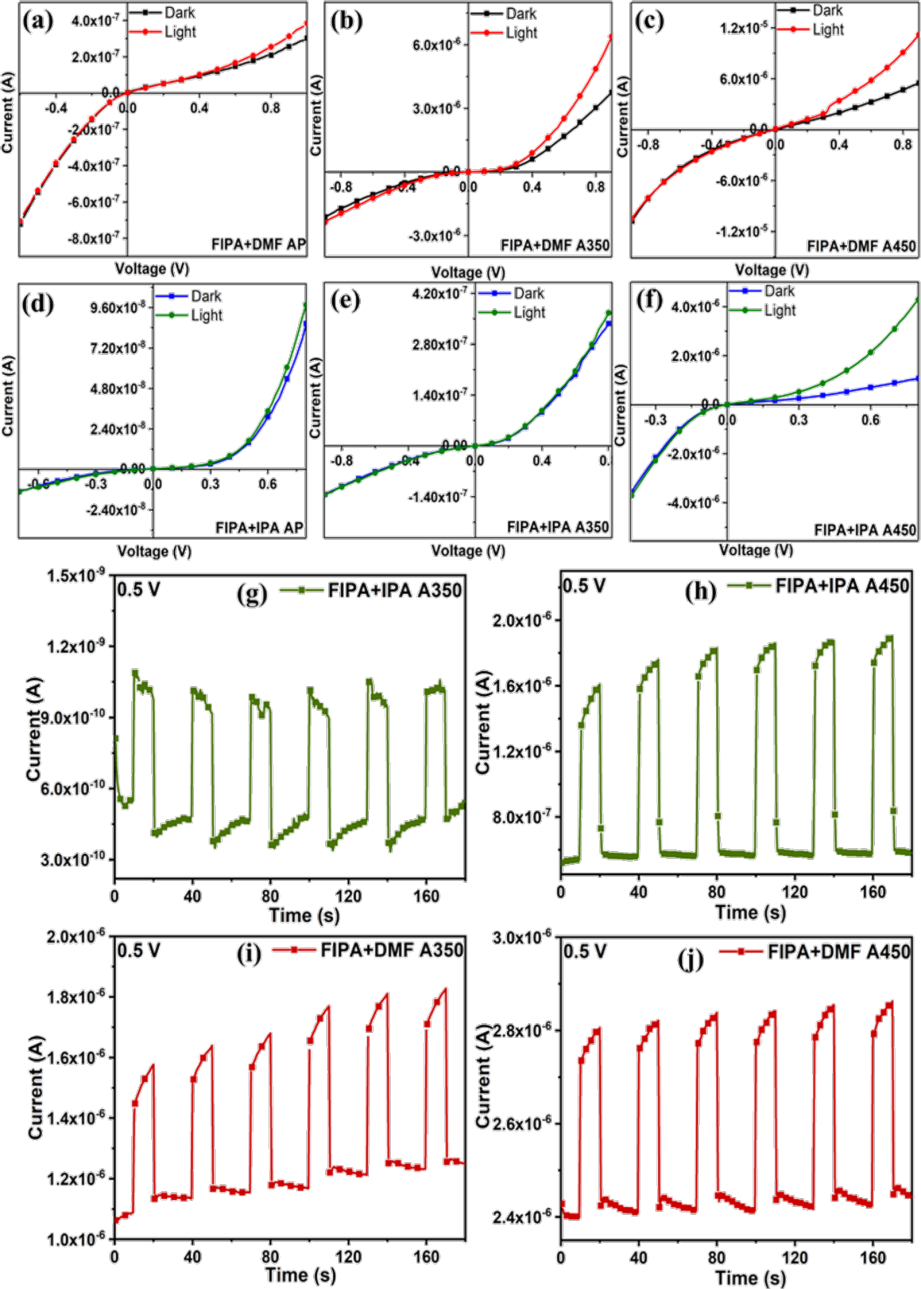
(a–f) *I*–*V* characteristics of the p-FeS_2_/n-Si photodiode – as prepared (AP), annealed at 350 °C (A350), and 450 °C (A450), (g–j) cyclic photoresponse measurements of p-FeS_2_/n-Si photodiode under illumination using tungsten lamp and a bias voltage of 0.5 V.

A comprehensive analysis on optoelectronic properties of the films were conducted on the fabricated device. The diode characteristics are presented in Figure 10a–f for as-prepared and annealed samples. It is important to note that all these fabricated devices (as prepared and annealed) as diodes exhibit fast photoresponse under light, as illustrated in Figure 10. Additionally, the exponentially sharp increase in current values with respect to the applied voltage under illumination also suggests that p-FeS_2_/n-Si diodes are photoconductive in nature. As there was not much change in the value of current obtained for films in as-prepared conditions, those samples were not included in further studies. Figure 10g–j illustrates the cyclic on/off behavior of the p-FeS_2_/n-Si diode using an illumination from tungsten (W) at a bias voltage of 0.5 V. In contrast to the dark condition, under illumination, the diode generates its maximum amount of charge carriers, which increases the current value. Based on these findings, the p-FeS_2_/n-Si diode has an excellent photosensitive nature, and it may be employed for optoelectronic applications. The morphological variations between the two sets of spin-coated films are primarily responsible for the variance in photocurrents between them. In the case of FIPA spin-coated films, charge recombination at the grain boundaries, due to the spherical and rod-shaped surface, may be the cause for comparatively reduced photocurrent. Additionally, it has been reported in CZTS thin films that the electron mobility of spherical and rice-shaped nanoparticles exhibits comparable values (≈430 cm^2^·V^−1^·s^−1^) and is greater than that of rod-shaped particles (≈260 cm^2^·V^−1^·s^−1^). The combination of rice-like shaped particles with sphere-like particles as in FDMF spin-coated films (in our case) are intriguing because the rice-shaped NPs probably have a less dense capping layer at the sharp apex, which makes carrier transfer easier [61].

**Figure 11:**
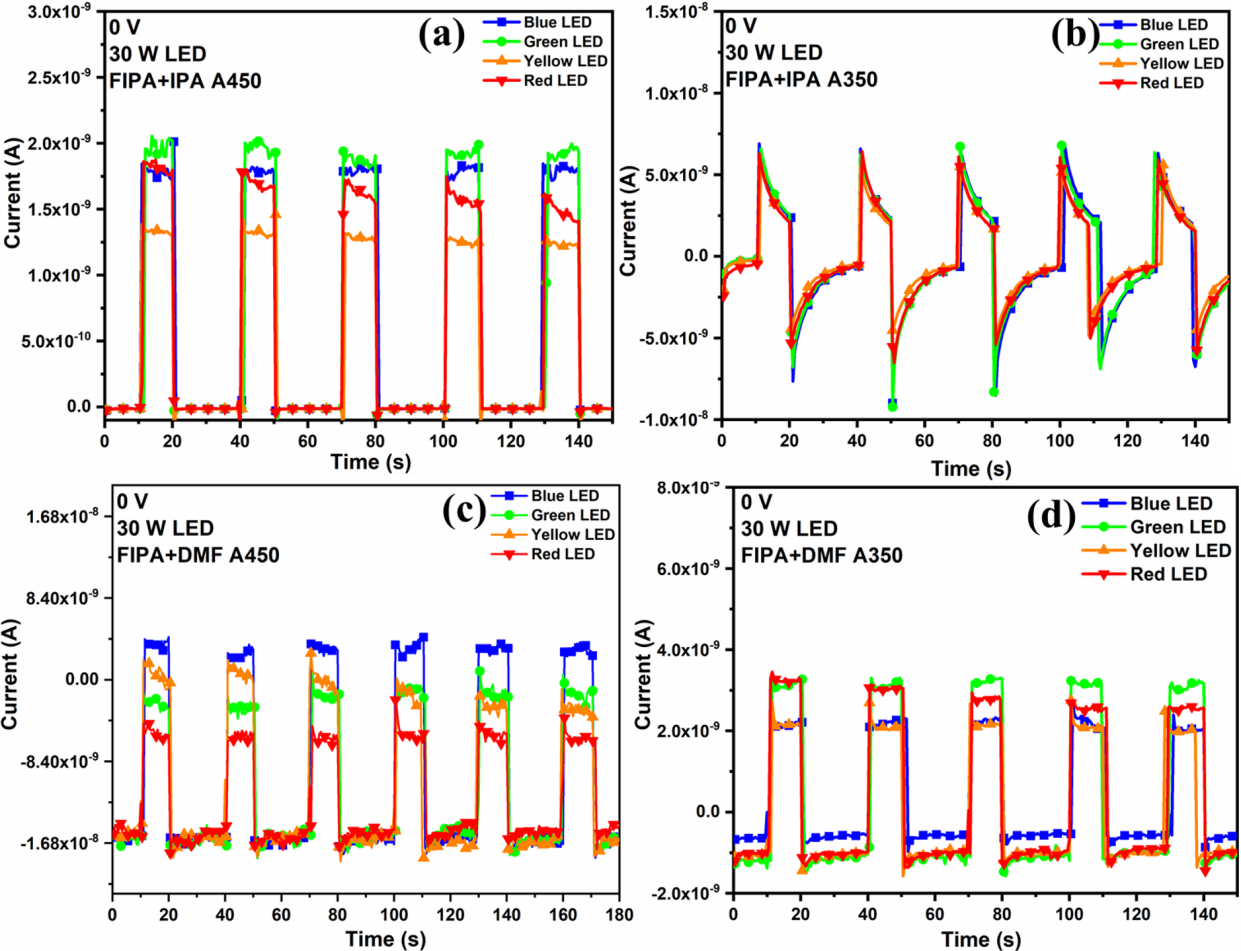
(a–d) Photoresponse measurements of p-FeS_2_/n-Si photodiode under illumination using different wavelengths of LEDs under self-powered mode.

Due to their benefits of high sensitivity, device downsizing, and low power consumption, self-powered photodetectors, which have numerous uses in the military, civilian purposes, and notably those related to green technology and flexible electronics have been increasingly popular in recent years [33,62]. This greatly interested us in this area and motivated to further look into its possibilities. The fabricated diodes were illuminated using W lamp (results are given in Supporting Information File 1, Figure S6), different wavelegths of LEDs (Figure 11) without applying any voltage and all the diodes showed excellent reponse. The peak wavelengths of LEDs were 465, 520, 590, and 620 nm for blue, green, yellow, and red LEDs, respectively. In the self-powered mode, spikes and overshoots can be seen in the transient photocurrent response of FIPA+IPA A350 film (Figure 11b and Supporting Information File 1, Figure S6b), which are typically interpreted as indicators of surface recombination. Peter et al. have explained this mechanism in semiconductor photoanodes [63]. When light is turned on, an immediate photocurrent appears due to the rapid separation of electron–hole pairs in the space charge region. This initial response is followed by a decay phase, where the accumulation of minority carriers near the interface leads to recombination and electron flow into the surface. Over time, a steady state is reached when the rate of hole influx at the interface balances charge transfer and recombination, resulting in a stable photocurrent. When the light is turned off, the process stops, and only residual electron movement remains. As the electron flux decays, any leftover holes are consumed by recombination and charge transfer, causing the current to return to zero with a negative overshoot. This justification suggests that since both the instantaneous negative spike and the reduction in photocurrent until the point of light switch off measure the recombination current, they should be of similar magnitude. The steady-state photocurrent (as in other films Figure 11a,c,d), in contrast, is the flux of holes that successfully enter the interface without coming into contact with any surface electrons [64]. In summary, self-powered photodetectors operate based on the photovoltaic effect in semiconductors, where incident light generates electron–hole pairs. The resulting photocurrent arises from the separation and directs movement of these charge carriers. In p-n junction photodetectors, an internal electric field further facilitates this separation, enhancing the response of the device [65].

**Figure 12:**
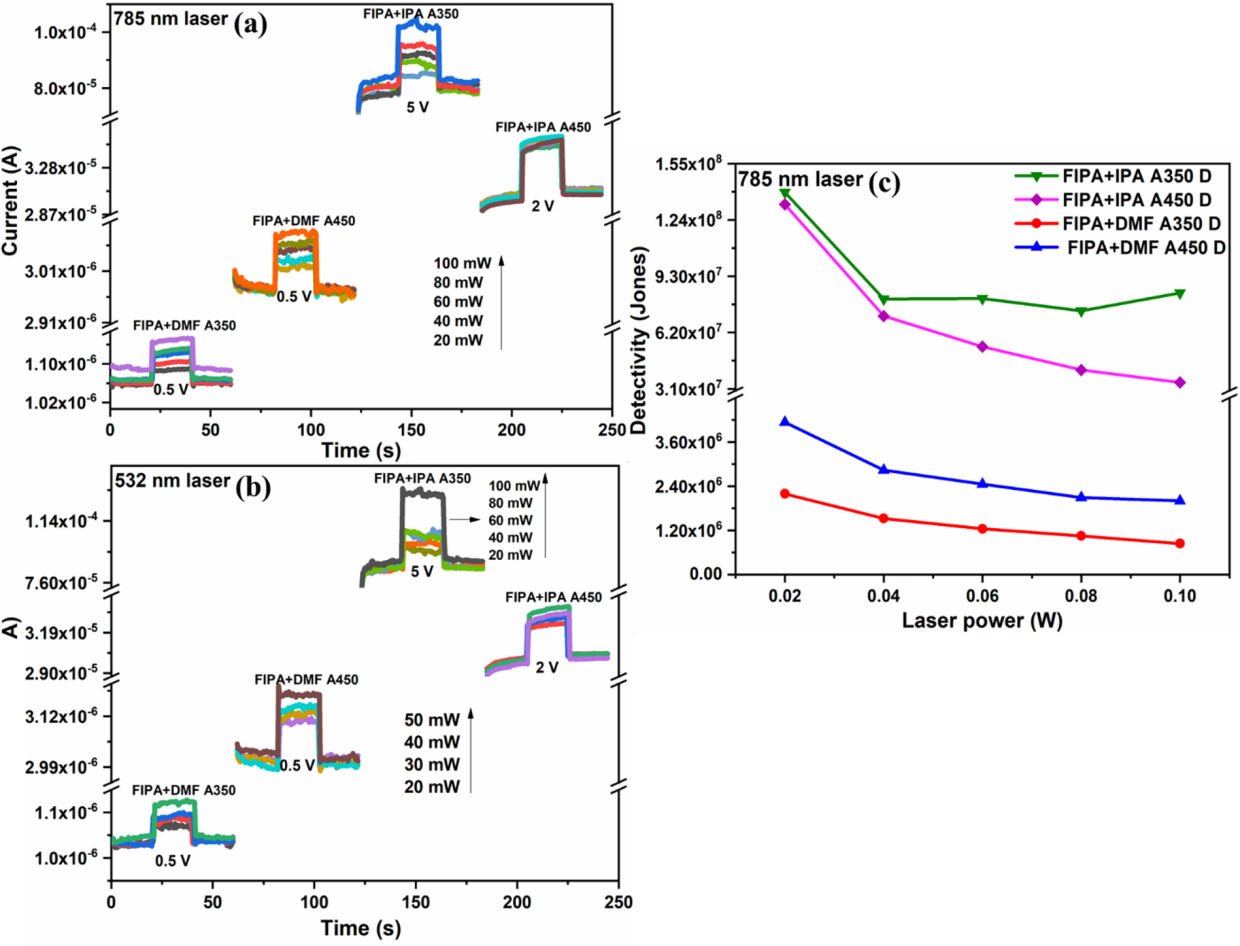
(a, b) Photoresponse measurements of p-FeS_2_/n-Si photodiode under illumination using continuous lasers of wavelength 785 and 532 nm respectively. (c) Detectivity of p-FeS_2_/n-Si photodiode under illumination using continuous laser of wavelength 785 nm.

The effect of light intensities at different wavelengths was also investigated while illuminating using 532 and 785 nm continuous laser source with variable output power. The power of the source was changed from 20 to 100 mW in all cases for the 785 nm continuous laser, and the applied voltages in each sample are also shown in Figure 12a. For the 532 nm laser, with the exception of FIPA (spin-coated film that was annealed at 350 °C), all other samples were illuminated with the power adjusted from 20 to 50 mW with a 10 mW increment every time. The increment for FIPA+IPA A350 was 20 mW each time. Figure 12b shows the response obtained for these measurements.

The quantity of photocurrent produced per unit power of the incident light on an effective area is known as spectral responsivity (*R*), and the evaluation of the least detectable radiant power, the normalized specific detectivity (D*), were evaluated by illuminating the photodiode using a 785 nm laser. The responsivity and detectivity were calculated using the Equations 1 and 2 [66]:


[1] $$R=\frac{I_{\text{light}}-I_{\text{dark}}}{PS},$$

[2] $$D*=\frac{R\sqrt{S}}{\sqrt{2eI_{\text{dark}}}},$$

where *R* is the spectral responsivity, *S* is the active area of illumination (0.7853 cm^2^), *e* is the electronic charge, *I*_light_ and *I*_dark_ are the current values under light and dark, and *P* is the optical power density. The optical power density values for 585 and 785 nm lasers, ranging from 20 mW to 100 mW, were between 0.070 and 0.3536 W/cm^2^. The detectivity and responsivity plot is given in Figure 12c and Supporting Information File 1, Figure S7 respectively. For FIPA spin-coated films, values of detectivity were in the order of 10^8^ Jones, and the values of responsivity were in the order of 10^−3^ A·W^−1^. The detectivity and responsivity for FDMF coated films were in the range of 10^6^–10^7^ Jones and 10^–6^–10^–5^ A·W^−1^, respectively. The responsivity and detectivity are slightly dropped as laser power increased, showing that the device is most effective at lower laser powers. The presence of trap states [67] in FeS_2_ can be used to explain this trend. These results are comparable to the reports by Zebarjad et al. on pyrite-based photodetectors. The detectors were developed by electrochemical deposition utilizing chemical precursors, where they stated FeS as the main phase and carried out sulphurization to obtain phase-pure FeS_2_. A photodetector with detectivity of around 10^8^ Jones was reported [68]. Mohsin et al. reported an external magnetic field-assisted laser ablation in liquid technique to prepare FeS_2_ nanoparticles [38]. They reported fabrication of FeS_2_ NPs/p-Si photodetectors using dipping technique, which demonstrated a detectivity of 10^11^ Jones. Phase-pure pyrite nanocrystals produced using a hydrothermal polymer-assisted technique had a broad photoresponse in the UV–vis spectral region, with normalized photocurrents as high as 1 to 100 A·cm^–2^ when a voltage between 0 and 3 V was applied [69]. In another study, the optoelectronic properties of FeS_2_ films in the visible light spectral region were made by doping and undoping Zn onto electrodeposited pyrite films. The Zn-doped samples exhibited a 9.2-fold increase in responsivity. The best Zn-doped condition showed a responsivity and detectivity of 0.206 A·W^–1^ and 3.3 × 10^9^ Jones, respectively, while a detectivity of 1.98 × 10^9^ Jones was reported for undoped samples, which are analogous to the values obatined in present study. This results also suggests a future possibility of doping the films prepared using laser ablated nanocolloids for refining its photodetector performances [70].

In this article, the generation of pyrite nanoparticles of different morphologies by laser ablation in different solvents was studied. As seen in this study, spherical and rod-like forms in FIPA, spherical with rice-grain morphology in FDMF and only spherical shapes in FAC, FMET, and FET are observed. A similar effect was observed in studies of Cu NPs, where spherical shapes were seen in deionized water, ethanol, and methanol, while rod-like morphologies were observed when methanol was used as the solvent [71]. Therefore, the solvent properties and their interactions with the material (FeS_2_ in this case) play a significant role in morphology. From the results obtained, it can be concluded that high-polarity solvents (e.g., methanol, ethanol) tend to promote faster nucleation and growth, leading to larger NPs (≈26 nm), whereas low-viscosity and highly volatile solvents (e.g., acetone) allow for rapid NP formation. This can result in bimodal size distributions (with smaller nanoparticles (1–35 nm) and some larger particles (50–60 nm)) with an average size of 17.06 nm due to uneven growth conditions, which may lead to broader size distributions or agglomeration. Solvents with better stabilization properties, such as moderate polarity and viscosity (e.g., IPA) help in the control of nucleation, growth, cooling rate, and aggregation of nanoparticles. The relatively narrow size distribution suggests that IPA provides a balanced environment for the formation and stabilization of FeS_2_ NPs, although bimodal distributions are observed (1–50 and 81–85 nm, with an average of 19.91 nm). For FeS_2_ in DMF (FDMF), the lack of discernible particles is attributed to the poor evaporation rate of the solvent, which prevents rapid cooling and particle formation, hindering size determination. High viscosity and boiling point of DMF hinder solvent evaporation during TEM sample preparation, making it difficult to discern the NPs. However, the high viscosity likely results in slower NP growth and potentially smaller sizes.

**Figure 13:**
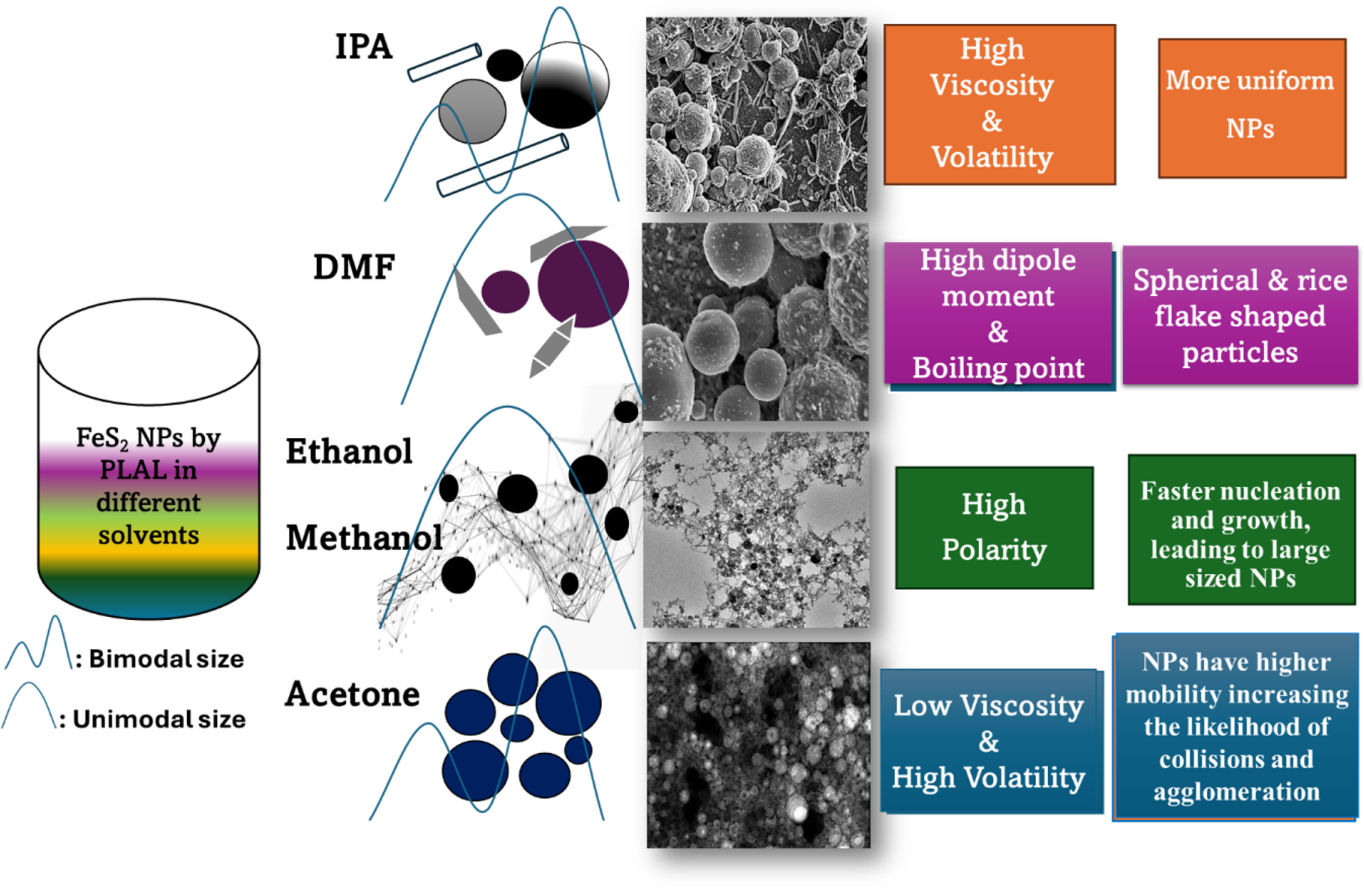
Schematic representation of FeS_2_ nanoparticles dispersed in different solvents, illustrating solvent-dependent transformations or products.

These variations in size highlight the role of the solvent in stabilizing nanoparticles and influencing their growth, with solvent viscosity, polarity, and evaporation rate being key factors in controlling nanoparticle size during PLAL synthesis [72]. A schematic representation of FeS_2_ nanoparticles dispersed in different solvents, illustrating solvent-dependent transformations, are given in Figure 13. The results observed are contrary to those reported for generation of Ag NPs in different solvents [42]. They reported that acetone and water restricted the growth mechanisms and small narrow NPs of Ag were observed. In our study, though the average size of ≈17 nm for FeS_2_ NPs obtained in acetone is comparable to the reported work on Ag, we observed precipitation within a few minutes. The precipitation of FeS_2_ NPs in acetone can be attributed either to the relatively low viscosity (0.306 mPa·s) of acetone, which means that NPs have higher mobility, increasing the likelihood of collisions and agglomeration if the repulsive forces are insufficient compared to other solvents, or the material-specific properties of FeS_2_, such as its semiconductor nature, weaker surface charge, and potential surface reactivity [73]. These factors reduce the effectiveness of electrostatic or steric stabilization, leading to agglomeration and precipitation. This behavior contrasts with metallic Ag NPs, which remain stable in acetone due to stronger electrical double layers and surface charge. According to the literature, in the case of nanosecond (ns) laser pulses, the laser energy can further excite the plasma plume, which is believed to promote homogenization of the ablated material. This effect often results in narrower nanoparticle size distributions rather than larger particles [37]. In the reported work also, no significant agglomeration was observed in the colloids, except for FeS_2_ NPs in acetone where sedimentation or settling down of particles was noted within a few hours, which is primarily due to the lower colloidal stability in acetone as explained above, rather than being a direct consequence of the nanosecond ablation process.

Pyrite (or fool’s gold) is one of the most appealing materials in terms of price and abundance, and was listed as one of the top 23 most promising choices for solar electricity production. Using nanocolloids prepared by PLAL, semiconducting pyrite thin films are prepared and characterized for photodetector application having good light absorption properties. Also, self-powered thin film photodiodes were constructed based on nanostructured pyrites. The outcome of this study ought to be helpful in research on synthesis of pyrite NPs by PLAL and their thin films for more optoelectronic device applications. Here, we anticipate more advancements in self-powered photodetectors based on the reported photodiodes of pyrite.

## Conclusion

In this work, the production of pyrite nanoparticles was achieved by a "green" synthesis technique, PLAL in different solvents. Ablation performed on various liquid media having varying thermodynamic properties influenced the formation of nanoparticles with various sizes and morphologies. Morphologies, including rod-shaped, spherical, and spherical particles joined by chains, were produced. The bandgap of the pyrite NPs varied between 3.0–3.6 eV in different solvents with values of 3.01, 3.12, 3.06, and 3.6 eV for NPs in IPA, DMF, methanol, and ethanol, respectively. The generated pyrite nanoparticles had features helpful for their usage in significant devices. Though the presence of FeS phase was identified along with pyrite NPs, by sulfurization process, phase-pure pyrite films were obtained. By a combination of EPD and spin-coating techniques, photodiodes were fabricated on n-type silicon substrates. Spin-coated films prepared with FeS_2_ NPs in DMF with scattered rice-like morphology demonstrated a greater photoresponse than spin-coated films using FeS_2_ in IPA. The photodiode showed a detectivity of 10^8^ Jones and a responsivity of 10^−3^ A·W^−1^ for FeS_2_ films prepared using NPs in IPA. To the best of our knowledge, there are no previous reports on PLAL-synthesized FeS_2_ in different liquids, and this is the primary results of self-powered photodetectors based on pyrite thin films. The results obtained could have significant implications for future optoelectronic and solar energy applications of pyrite materials.

## Supporting Information

Images of the FeS_2_ nanocolloids prepared in different solvents, tabulation of physical properties of the solvents and binding energy values of the elements from XPS, optical studies of FeS_2_ nanoparticles, XRD, SEM and photoresponse measurements of the pyrite nanoparticles and thin films.

File 1 Additional tables and figures.

## Data Availability

Data generated and analyzed during this study is available from the corresponding author upon reasonable request.
