# Supplementary material for: Morphology and properties of pyrite nanoparticles obtained by pulsed laser ablation in liquid and thin films for photodetection
Source: Beilstein J Nanotechnol. 2025 Jun 3;16:785–805. doi: 10.3762/bjnano.16.60 (PMC12152315; doi:10.3762/bjnano.16.60)
Supplement: File 1 — Additional tables and figures. [file Beilstein_J_Nanotechnol-16-785-s001.pdf]

BEILSTEIN JOURNAL OF NANOTECHNOLOGY

Supporting Information

for

Morphology and properties of pyrite nanoparticles obtained by pulsed laser ablation in liquid and thin films for photodetection

Akshana Parameswaran Sreekala, Bindu Krishnan, Rene Fabian Cienfuegos Pelaes, David Avellaneda Avellaneda, Josué Amílcar Aguilar-Martínez and Sadasivan Shaji

Beilstein J. Nanotechnol. **2025,** *16,* 785–805. doi:10.3762/bjnano.16.60

Additional tables and figures

License and Terms: This is a supporting information file under the terms of the Creative Commons Attribution License (https://creativecommons.org/licenses/by/4.0). Please note that the reuse, redistribution and reproduction in particular requires that the author(s) and source are credited and that individual graphics may be subject to special legal provisions.
The license is subject to the *Beilstein Journal of Nanotechnology* terms and conditions: (https://www.beilstein-journals.org/bjnano/terms)

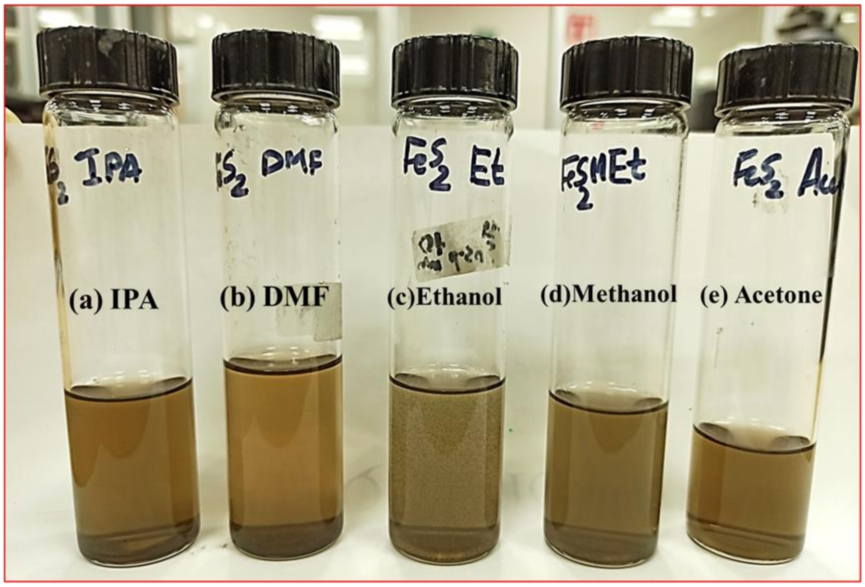

Figure S1: Images of FeS_2 nanocolloids prepared in different solvents (FIPA, FDMF, FET, FMET, and FAC respectively).

Table S1: Physical properties of the solvents used for the synthesis of pyrite NPs [1-5].

Solvents	Boiling point (°C)	Dipole moment (D)	Dielectric constant	Density (kg/m^3)	Viscosity (mPa s)	Vapor Pressure (Torr)
Isopropyl alcohol (IPA)	82.5	1.66	17.9	781	2.5	15
Dimethyl formamide (DMF)	153	3.86	36.71	944	0.796	2.7
Ethanol	78.3	1.69	24.5	789	1.074	43.9
Methanol	64.7	1.70	32.7	792	0.543	97
Acetone	56	2.88	20.7	784	0.306	184.5

Table S2: Binding energy values of the elements (Fe 2p, S 2p) identified in FeS_2 NPs synthesized in different solvents.

**Samples**	**Binding energy (eV)**						
	**Fe^{2+} in FeS_2**		**Fe(II)–S in FeS**	**S^{2-} in FeS**		**S_2^{2-} in FeS_2**	
	**Fe 2p $_{3/2}$**	**Fe 2p $_{1/2}$**		**S 2p $_{3/2a}$**	**S 2p $_{1/2a}$**	**S 2p $_{3/2b}$**	**S 2p $_{1/2b}$**
**FIPA**	709.68	722.78	713.35	161.15	162.33	161.42	163.6
**FDMF**	709.17	722.27	713.96	161.84	163.02	162.96	164.14
**FET**	709.47	722.57	714.11	161.22	162.4	162.45	163.36
**FMET**	709.88	722.98	712.49	161.66	162.84	162.42	163.6
**FAC**	709.55	722.65	713.85	161.28	162.46	162.02	163.2

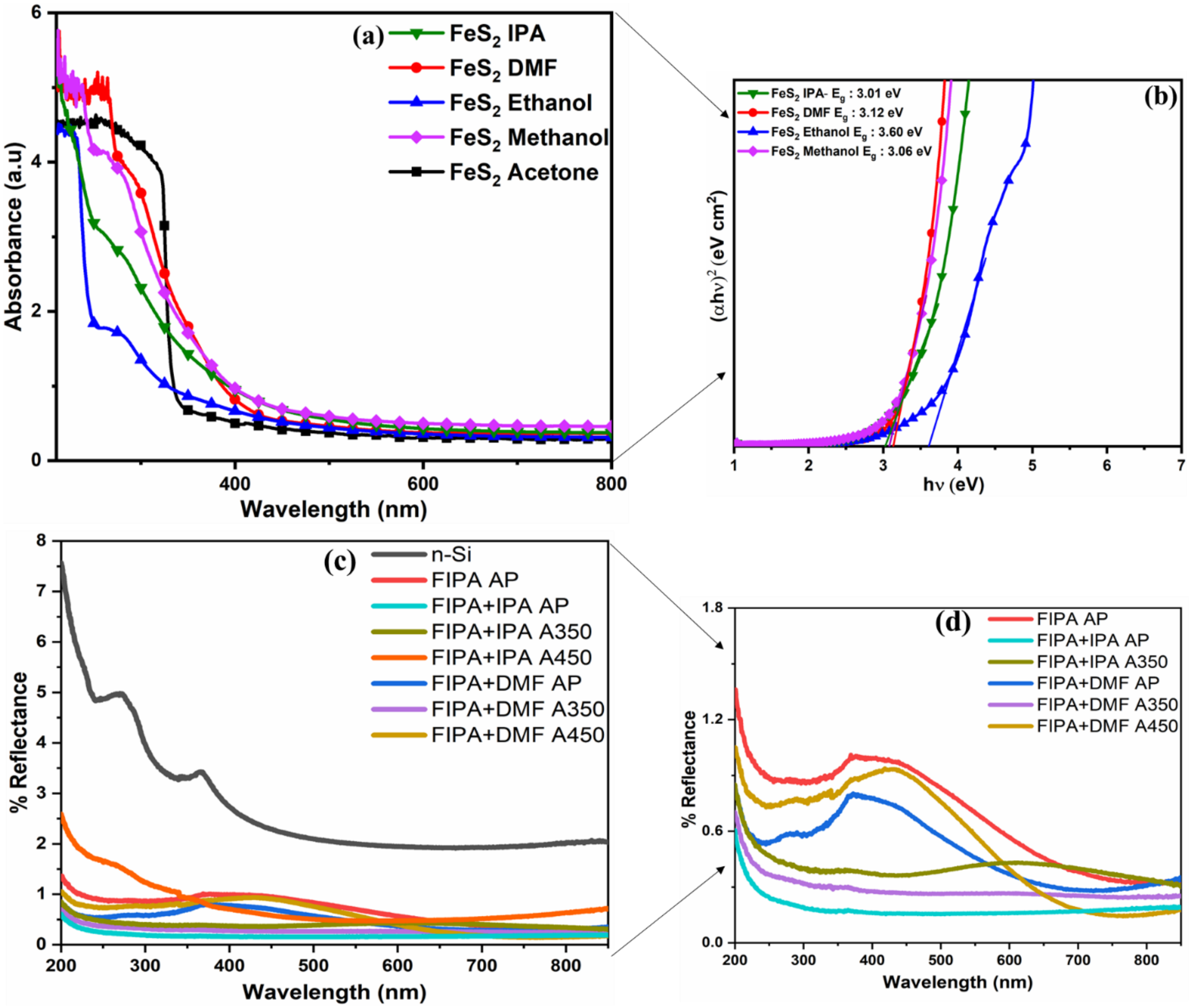

Figure S2: (a) The absorption spectra of FeS_2 nanoparticles synthesized by PLAL in different solvents (b, c). % Reflectance of as-prepared and annealed films, (d) zoomed image of the % reflectance of the films.

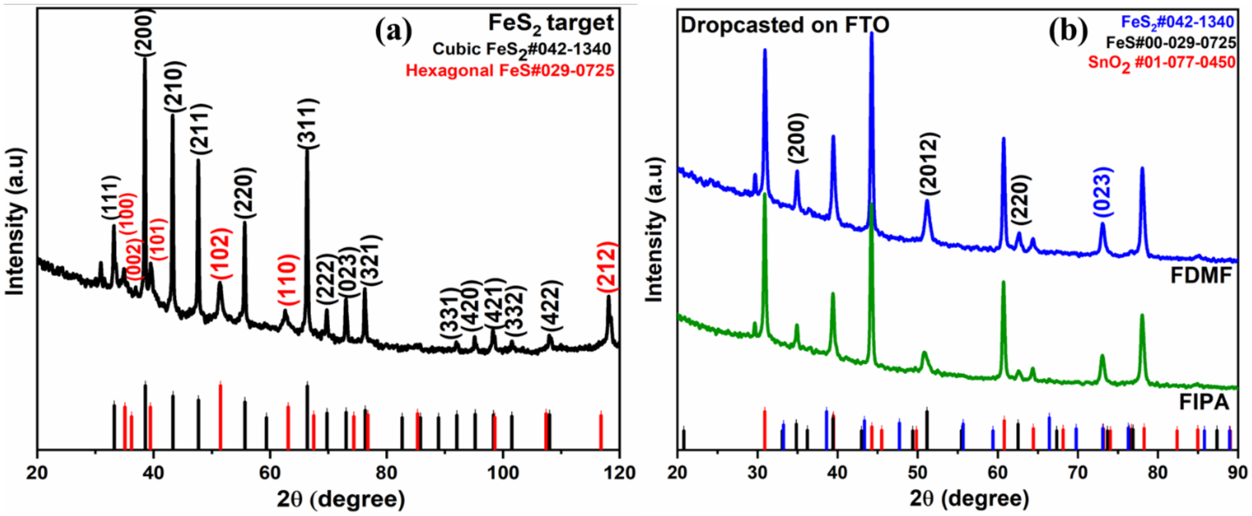

Figure S3: XRD patterns of the (a) pyrite target used for PLAL – diffraction peaks for FeS_2 and FeS are noted. (b) Films of FIPA and FDMF drop casted on FTO substrates.

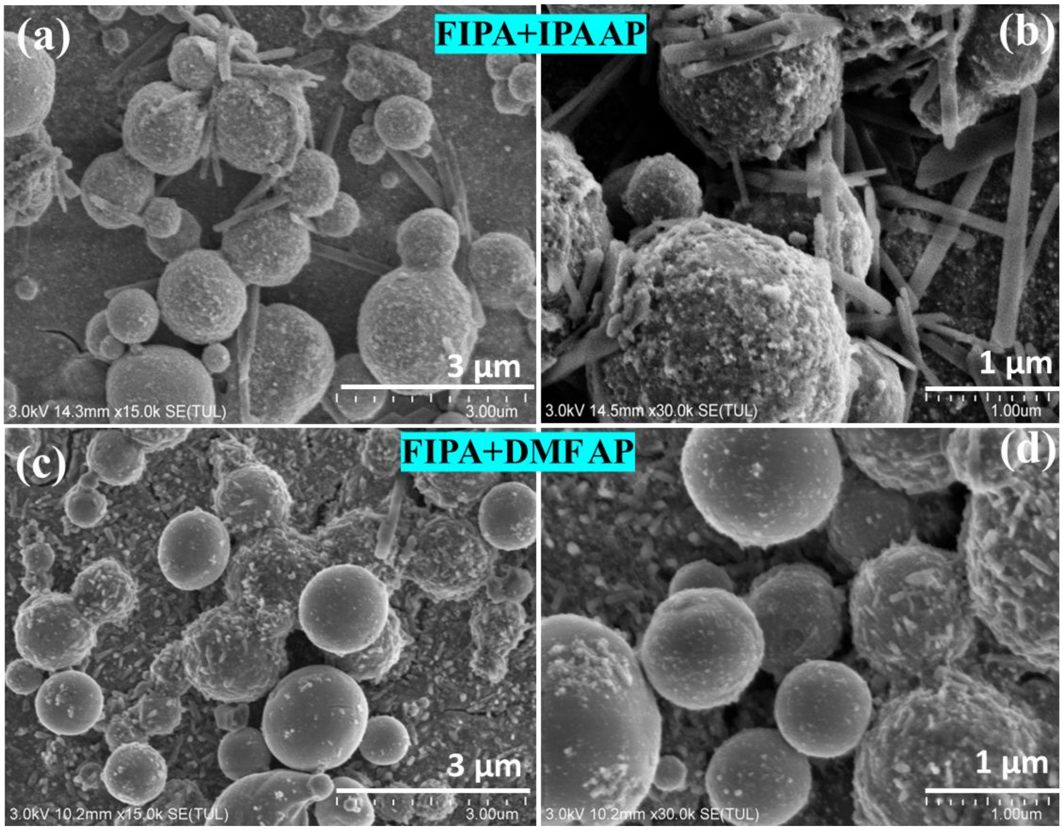

Figure S4: SEM images of as-prepared thin films fabricated on n-Si by EPD above which (a, b) FeS_2 NPs in IPA (FIPA) and (c, d) FeS_2 NPs DMF (FDMF) are spin coated.

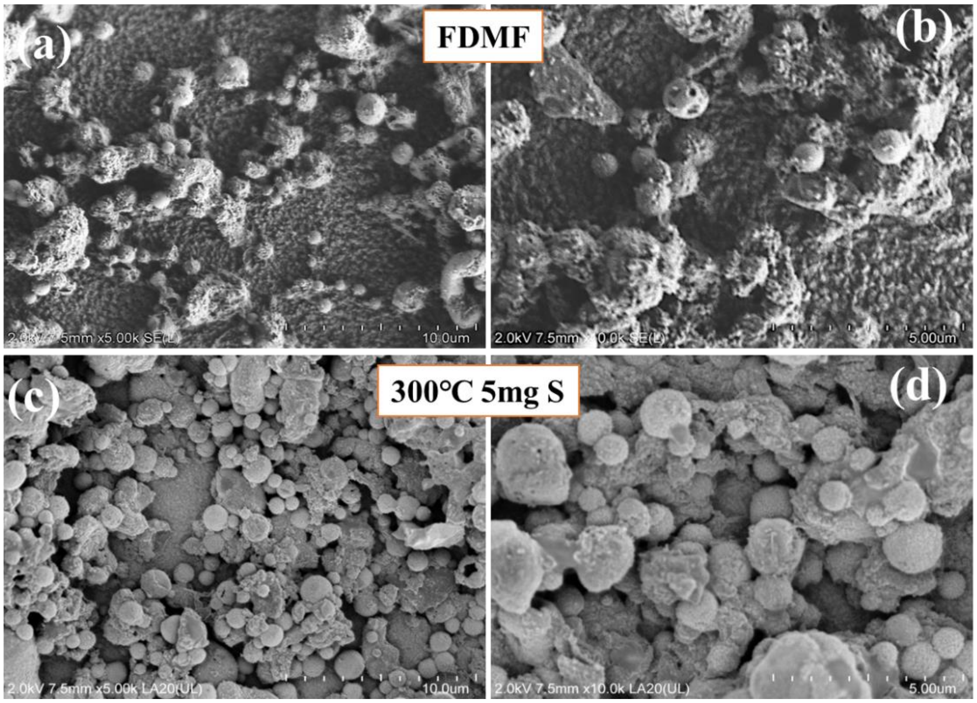

Figure S5: SEM images of as-prepared FDMF films on FTO (a, b) before and (c, d) after sulfurization. (Image scales are 10 and 5 μm).

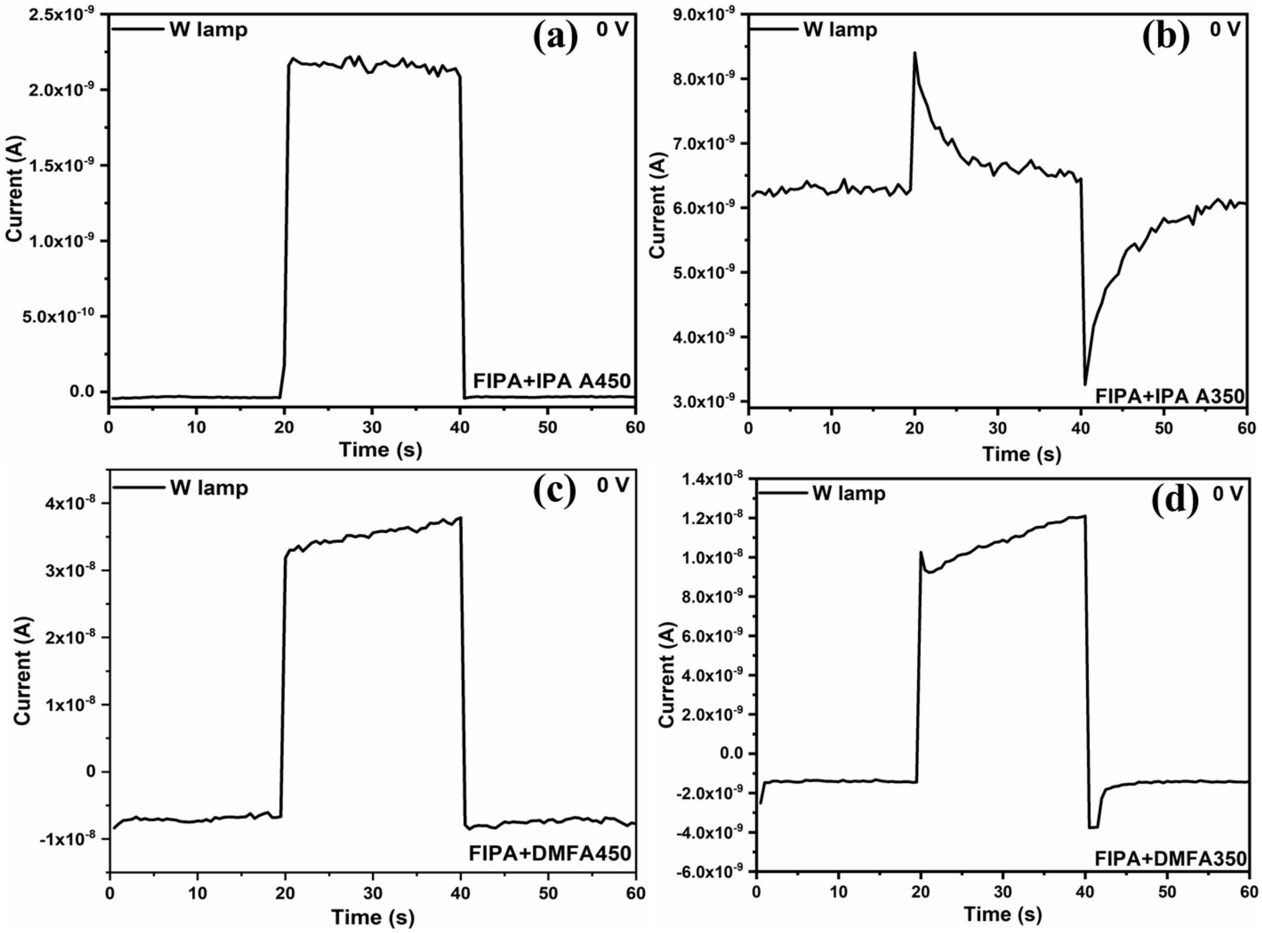

Figure S6: Photoresponse measurements of p-FeS_2/n-Si photodiode under illumination using W lamp under self-powered mode.

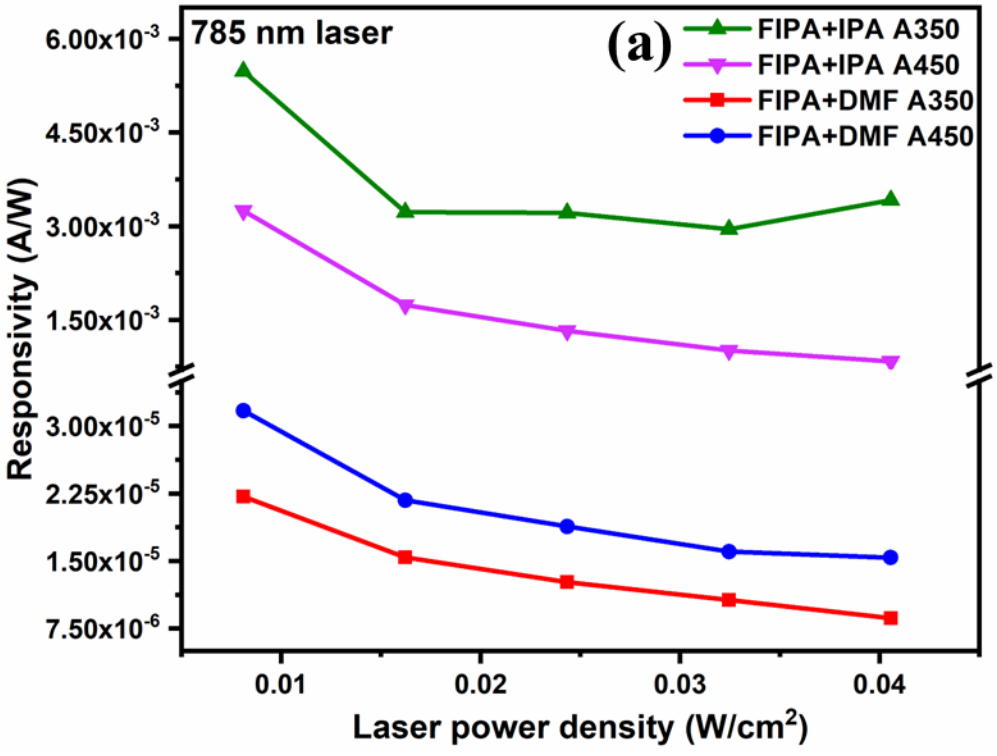

Figure S7: Responsivity of p-FeS_2/n-Si photodiode under illumination using 785 nm laser.

References

(1) Israelachvili, J. N. *Intermolecular and Surface Forces*, 3rd ed.; Diego, A. P. S., Ed.; Academic Press: Burlington, MS, 2011. doi:10.1016/C2009-0-21560-1

(2) Yao, L.; Gerde, J. A.; Wang, T. *J. Am. Oil Chem. Soc.* **2012**, *89*, 2279–2287. doi:10.1007/s11746-012-2124-9

(3) Sigma-Aldrich. Physical Properties of Solvents, Lab Basics Handbook, p 144; Sigma-Aldrich https://www.sigmaaldrich.com/deepweb/assets/sigmaaldrich/marketing/global/documents/614/456/labbasics_pg144.pdf?srsltid=AfmBOopQSD5UiPLXeE39xtg5uhn3DblzhnRXIMJMnivgauhdgDcoZJom (accessed Jan 15, 2025)

(4) Li, C.-P.; Du, M. *Chem. Commun.* **2011**, *47*, 5958. doi:10.1039/c1cc10935a

(5) Yang, C.; Sun, Y.; He, Y.; Ma, P. *J. Chem. Eng. Data* **2008**, *53*, 293–297. doi:10.1021/je700430g
